# Goal-directed behavior and hippocampal activity predict real-life impact of drinking intentions in alcohol use disorder

**DOI:** 10.1038/s41398-025-03660-5

**Published:** 2025-10-20

**Authors:** Claudia Ebrahimi, Milena P. M. Musial, Nuria Doñamayor, Diana S. Prychynenko, Erik L. Bode, Rainer Spanagel, Andreas Heinz, Lorenz Deserno, Michael N. Smolka, Ulrich Ebner-Priemer, Reinhold Kliegl, Tanja Endrass, Markus Reichert, Michael Rapp, Florian Schlagenhauf

**Affiliations:** 1 Charité – Universitätsmedizin Berlin, corporate member of Freie Universität Berlin and Humboldt-Universität zu Berlin, Department of Psychiatry and Neurosciences | CCM, NeuroCure Clinical Research Center, 10117 Berlin, Germany; 2 Institute of Clinical Psychology and Psychotherapy, Faculty of Psychology, Technische Universität Dresden, 01062 Dresden, Germany; 3 Humboldt-Universität zu Berlin, Faculty of Life Sciences, Department of Psychology, 10099 Berlin, Germany; 4 Charité – Universitätsmedizin Berlin, corporate member of Freie Universität Berlin and Humboldt-Universität zu Berlin, Einstein Center for Neurosciences Berlin, 10117 Berlin, Germany; 5 Charité – Universitätsmedizin Berlin, corporate member of Freie Universität Berlin and Humboldt-Universität zu Berlin, Bernstein Center for Computational Neuroscience, 10117 Berlin, Germany; 6 Institute of Psychopharmacology, Central Institute of Mental Health, Medical Faculty Mannheim, University of Heidelberg, 68159 Mannheim, Germany; 7 German Center for Mental Health (DZPG), partner site Mannheim/Heidelberg/Ulm, Germany; 8 Department of Psychiatry and Psychotherapy, Universität Tübingen, 72076 Tübingen, Germany; 9 Department of Psychiatry and Psychotherapy, Technische Universität Dresden, 01062 Dresden, Germany; 10 Department of Child and Adolescent Psychiatry, Psychosomatics and Psychotherapy, University Hospital Würzburg, 97080 Würzburg, Germany; 11 Mental mHealth Lab, Department of Applied Psychology, Institute of Sports and Sports Science, Karlsruhe Institute of Technology, 76131 Karlsruhe, Germany; 12 Department of Psychiatry and Psychotherapy, Central Institute of Mental Health, Medical Faculty Mannheim, University of Heidelberg, 68159 Mannheim, Germany; 13 Social and Preventive Medicine, University of Potsdam, 14476 Potsdam, Germany; 14 Department of Sport and Exercise Science, Faculty of Natural and Life Sciences, University Salzburg, 5020 Salzburg, Austria

**Keywords:** Learning and memory, Addiction

## Abstract

A shift away from goal-directed, model-based behavior is commonly viewed to characterize alcohol use disorder (AUD). Previous research, however, has failed to demonstrate differences between individuals with and without AUD regarding goal-directed control, operationalized as model-based behavior. Instead, findings suggest associations between model-based behavior and alcohol consumption patterns, but mechanistic insights into the link between model-based behavioral and neural signatures and longitudinal, real-life control over alcohol intake remain elusive. Here, we investigated whether experimentally assessed model-based behavior can prospectively predict intentional reduction of alcohol consumption in daily life. Therefore, we related behavioral and neural markers of model-based behavior during a sequential decision-making task in 67 participants with AUD (20 women) to long-term smartphone-based ecological momentary assessments of daily alcohol intake and weekly alcohol consumption intentions over a period of up to one year. Model-based behavior and its neural signatures in bilateral hippocampus and ventral striatum moderated how well individuals succeeded in aligning their alcohol consumption with their drinking intentions during the following year. Specifically, AUD participants with higher model-based behavior and associated stronger hippocampal and weaker ventral striatal learning signals exhibited enhanced capacity to intentionally reduce their alcohol consumption in everyday life. These findings provide evidence for the ecological validity of computational concepts of goal-directed behavior and suggest specific treatment targets for individually tailored interventions to regain control over alcohol use.

## Introduction

Eighty percent of the global population drink alcohol at some point during their life [1]. While most use alcohol only recreationally, some develop alcohol use disorder (AUD), which has a lifetime prevalence of 8.6% [1]. AUD is associated with reduced control over alcohol intake, which has been conceptualized in terms of instrumental learning: rewarding drug effects initially function as reinforcers of consumption behavior [2, 3]; however, through repetition, drug intake is thought to become more independent of the drug’s reinforcing effects and increasingly guided by drug-associated cues [2, 3].

Accordingly, one mechanism hypothesized to underlie the progression from recreational to excessive drug use is a shift from goal-directed to habitual behavior [2, 4–6]. Difficulties in implementing drinking reduction or cessation goals, as one aspect of loss of control over alcohol intake, indeed constitute a diagnostic criterion for AUD according to the Diagnostic and Statistical Manual of Mental Disorders (DSM)-5 [7]. Computationally, goal-directed and habitual behavior have been operationalized as model-based and model-free reinforcement learning, respectively, and are commonly assessed using sequential decision-making tasks. Here, participants repeatedly navigate through a decision tree with the explicit goal of maximizing their total reward [8–11]. A model-based strategy enables individuals to reach this goal in a flexible way by prospectively considering all available actions and their respective future outcomes, whereas a model-free strategy favors simple repetition of previously rewarded actions [12]. Both model-free and model-based learning have been reported to be encoded in ventral striatum (VS) [13] and hippocampus [14–17], while correlates of model-based behavior have additionally been observed in medial prefrontal regions [13].

Evidence from animal studies largely supports a shift from goal-directed to habitual control in the development of addiction-like behavior [18–20]. However, results in humans are inconsistent (for recent reviews, see [10, 11, 21]) and the concept of a shift towards habitual behavior has received considerable criticism and updates in recent years [22–26]. So far, findings do not suggest clear-cut differences between individuals with AUD or sub-clinical populations and healthy humans in probes of model-based control [27–29] or its neural correlates [28, 30]. Instead, model-based control has been associated with alcohol consumption and binge drinking trajectories in healthy young males [31] as well as abstinence duration in individuals with AUD [29]. Moreover, weaker model-based neural correlates in medial prefrontal cortex have been associated with a higher relapse probability [28]. However, no study to date has investigated the direct link between model-based behavior and day-to-day control over alcohol intake in non-abstinent individuals with AUD. This link seems plausible as goal-directed or model-based behavior is characterized by prospective planning and representation of the intended goal, which might, in turn, be needed to put drinking intentions into practice, i.e. to control alcohol intake.

The present study aimed to test the ecological and predictive validity of a laboratory two-step task [8, 32] regarding control over drinking in everyday life by relating model-based behavior and its neural correlates to the implementation of drinking intentions in non-treatment-seeking participants with mild to severe AUD. Sixty-seven individuals performed the two-step task during functional magnetic resonance imaging (fMRI) and completed ecological momentary assessments (EMA) of their prospective weekly intention to reduce consumption and their daily alcohol intake over a period of up to one year. Smartphone-based EMA has been shown to be very reliable for tracking real-life alcohol intake [33] and previous studies using EMA have successfully identified predictors of increased alcohol consumption [34, 35]. Here, we hypothesized that a higher degree of model-based behavior and of activation in brain regions encoding model-based reward prediction error (RPE) components would predict improved ability to implement intentions to reduce alcohol consumption in AUD.

## Methods and materials

### Participants, study design, and procedures

Ninety-three participants were recruited via public advertisement in Berlin and Dresden, Germany, as part of the Collaborative Research Center (CRC) 256 [36, 37]. Volunteers were excluded if they had an explicit desire for alcohol abstinence or if a clinical indication for medically supervised alcohol detoxification or acute therapeutic intervention was present. For complete exclusion criteria and statistical power considerations, see Supplementary Materials.

**Fig. 1:**
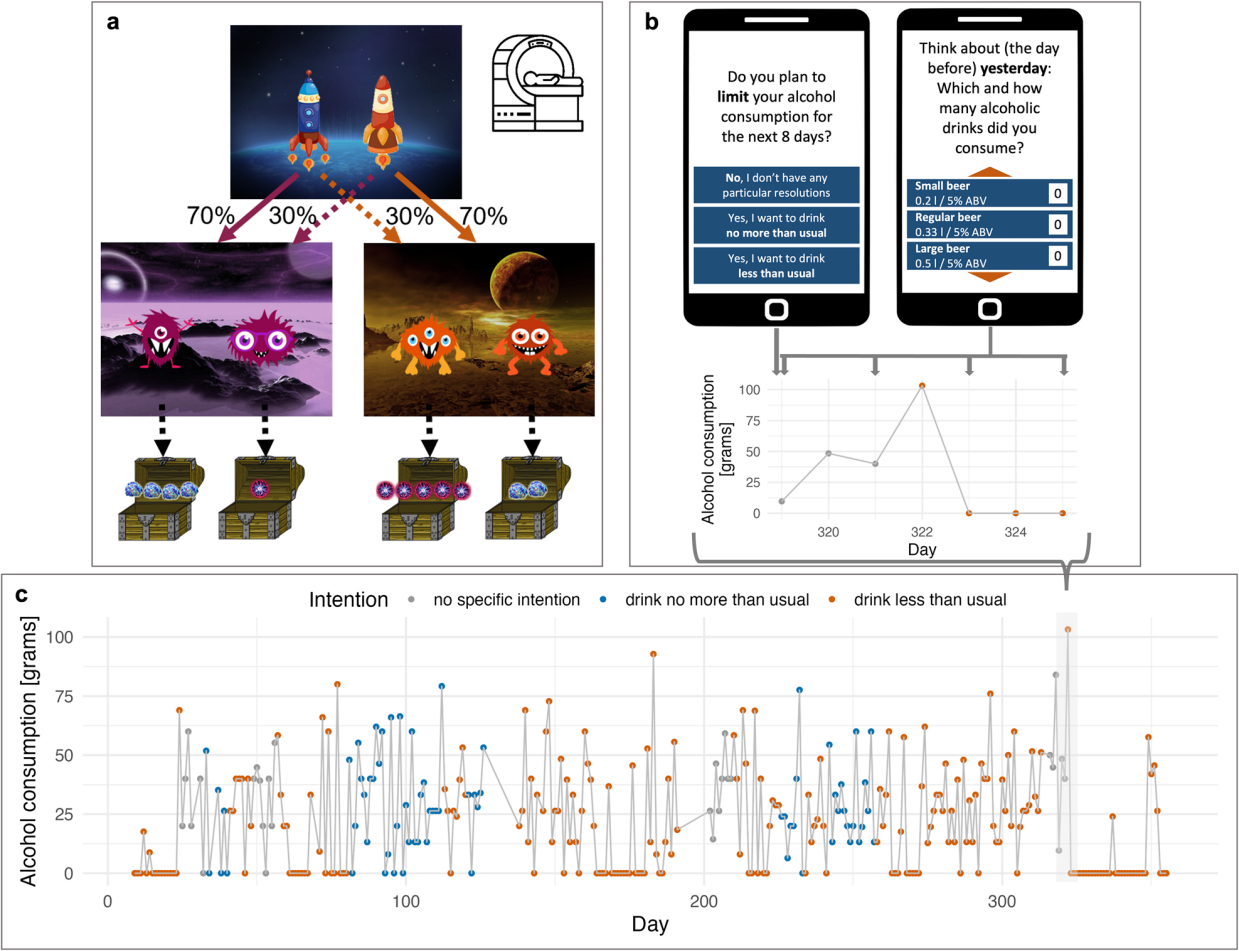
Study design and experimental procedures. **a** Symbolic representation of the two-step task adapted from Daw et al. [8] and Kool et al. [32] which was performed during fMRI at the beginning of the ecological momentary assessment (EMA) period. Each out of 201 trials started in a first state (planet earth), where subjects had to choose between two actions (spaceships). Each action led to one out of two second-stage states (planets) with a higher probability (common transition) than it led to the other second-stage state (rare transition). The two actions (aliens) available per second-stage state were rewarded or punished with points between +1 and +5 (comet dust, gold) and between -1 and -5 (anti-matter, black) according to Gaussian random walks, respectively (Supplementary Material). **b** Alcohol consumption trajectory over days with complete EMA data for one exemplary participant with AUD. Dot color reflects drinking intention assessed at the latest preceding point in time, respectively. **c** Drinking intention (left smartphone screen) and alcohol consumption (right smartphone screen) during an exemplary EMA week.

During an initial on-site assessment, all participants performed the two-step task [8, 32] (Fig. 1; Supplementary Material) during fMRI to assess the degree of model-based behavior, with 86 participants providing valid imaging data. Participants who fulfilled two or more AUD criteria (*n* = 67; 20 women, 47 men; age (*M* ± *SD*) = 35.2 ± 9.4 years) were followed up using EMA for up to 359 days to evaluate their weekly drinking intentions and their daily alcohol consumption (EMA sample; Figure S1). Of these, 21 participants were diagnosed with mild, 29 with moderate, and 17 with severe AUD (for sample characteristics, see Table S1). At the beginning of the assessment period, participants in the EMA sample additionally performed the Digit Symbol Substitution Test (DSST) [38], the Raven Standard Progressive Matrices [39], as well as the Digit Span Backwards Task (DSBW) [40].

### Ecological momentary assessment (EMA)

During the follow-up period, participants rated their retrospective alcohol consumption and their prospective drinking intention using a smartphone-based e-diary as they went about their daily routines. Alcohol intake was assessed every two days for each of the two past days separately: “Think about yesterday (*second item: the day before yesterday*): Which and how many alcoholic drinks did you consume?” (Fig. 1). Participants were asked to select from a list of alcoholic drinks with an indication of the amount of alcohol by volume and an input field to indicate the number of respective drinks consumed (Table S2). We restructured this variable, resulting in a continuous data set with a daily resolution, i.e., each entry contained the alcohol consumption in grams on one day.

Drinking intention was assessed every 8 days: “Do you plan to limit your alcohol consumption for the next 8 days?”. Possible answers were “No, I don’t have any particular intentions”, “Yes, I want to drink no more than usual”, and “Yes, I want to drink less than usual” (Fig. 1). We restructured drinking intention data so that each intention rating was assigned to the day it was queried and to the following 7 days. This resulted in a one-to-one mapping of drinking intention and alcohol intake per day. Days with missing alcohol intake and/or drinking intention EMA data were excluded from all analyses. For further details on EMA assessment, see Supplementary Material.

### Data analysis

Bayesian computational modeling of two-step data was performed in RStan [41, 42] and linear mixed models (LMMs) as well as logistic mixed-effects regression models (a special case of generalized LMMs [GLMMs]) were implemented using the lme4 [43] package in R, version 4.4.1 [44]. Robust re-estimation of LMMs was performed using the robustlmm [45] package. We estimated marginal means using the emmeans package [46]. Preprocessing and statistical analyses of fMRI data were performed within SPM12 (www.fil.ion.ucl.ac.uk/spm) implemented in MATLAB 2021b (The MathWorks, Inc., Natick, MA, USA). All results are reported on a two-sided significance level of *α* = 0.05.

#### Computational modeling and analysis of behavioral two-step data

Behavior in the two-step task can be formalized using model-based and model-free reinforcement learning algorithms [8]. Model-based learning, which is more computationally costly [9, 47], relies on a full internal model of the environment, i.e., it incorporates both the underlying transition structure from first- to second-stage states and received rewards [48]. In contrast, model-free learning favors simple repetition of previously rewarded actions, without considering the transition structure [9, 27]. We fitted a hybrid reinforcement learning model [8] to behavioral two-step data (for details on model comparison, see Supplementary Material). This model captures the relative balance between model-based and model-free behavior in the parameter *ω*, with *ω* = 1 indicating pure model-based and *ω* = 0 pure model-free control (Supplementary Material). Computational modeling considers the incremental trial-by-trial learning history of each individual and allows to derive model-based RPE components as a basis for subsequent fMRI analyses. To enhance comparability with previous sequential decision-making studies, we complemented our analyses by quantifying model-based vs. model-free behavior using logistic mixed-effects regression [29, 32, 49, 50], predicting the tendency to repeat or change the first-stage choice depending on information of the previous trial. The outcome (‘reward’ vs. ‘punishment’) × transition type (‘common’ vs. ‘rare’) interaction is considered indicative of model-based behavior (hereafter ‘MB score’, Supplementary Material) [8, 32].

To test whether model-based behavior is associated with AUD severity, we regressed the parameter ω and the MB score on dummy-coded AUD severity (‘moderate’ vs. ‘mild’, ‘severe’ vs. ‘mild’) using independent linear regression analyses. To test associations between model-based behavior and domain-general cognitive measures or the individual proportion of drinking reduction intentions, we computed Spearman’s rank correlation.

#### fMRI analysis

Imaging analyses are based on 86 participants with valid fMRI data (Figure S1; for fMRI acquisition and preprocessing, see Supplementary Material). First-level analyses were performed as previously described [8] (Supplementary Material). Specifically, a combined onset regressor for second stage and reward was parametrically modulated by (i) model-free RPEs and (ii) unique model-based RPE components, i.e., the difference between model-based and model-free RPEs. We computed first-level contrast images for both (i) and (ii) and used them for random-effects models at the group level. Study site was included as a second-level covariate. Model-free and model-based neural signatures were assessed with separate one-sample *t*-tests at a family-wise-error (FWE) whole-brain corrected significance level of *p* ≤ 0.05. We extracted mean values from bilateral vmPFC, VS, and hippocampus volumes of interest based on their involvement in forward planning and goal-directed behavior [8, 13–16]; Supplementary Material). To assess differences in model-based signatures depending on AUD severity, we conducted a full-factorial ANOVA with the difference regressor (ii) as dependent variable, a between-subject predictor AUD severity (‘mild’, ‘moderate’, ‘severe’), and study site as a covariate. The main effect of group was investigated at *p*_FWE_ ≤ 0.05 whole-brain corrected.

#### Linear and logistic mixed-effects models for real-life drinking behavior

To investigate whether the effect of weekly intentions to reduce consumption on daily alcohol intake in participants with AUD was moderated by the degree of model-based behavior in the two-step task and its neural correlates, we used LMMs with maximum likelihood estimation. Based on an unconditional LMM, we estimated an intra-class correlation coefficient of 0.16 for alcohol consumption, which could indicate that about 84% of variance was attributable to within-subject fluctuations (level 1 in our hierarchical statistical model).

We first set up a LMM using log gram alcohol/day (incremented by 1 to avoid zero scores) as dependent variable and including fixed effects for the computational parameter ω, drinking intention, and their interaction. Drinking intention was coded with two dummy contrasts using ‘no specific intention’ as reference category, i.e. contrast 1) compared ‘drink less than usual’ vs. ‘no specific intention’, and contrast 2) compared ‘drink no more than usual’ vs. ‘no specific intention’. We chose this coding scheme as our focus lay on contrasting time periods in which participants had the intention to reduce consumption with time periods with no such intention (i.e., the first contrast). We included the second contrast for completeness but had no corresponding hypothesis. The optimal random-effect structure in this model was identified via likelihood ratio test, comparing a LMM 1) estimating only a variance component for individual differences in mean log gram alcohol/day in the reference category (i.e. random intercept), 2) adding variance component estimates for individual differences in drinking intention (i.e. random slopes for contrasts 1 and 2), and 3) adding estimates for the correlation parameters between random intercept and random slopes. The likelihood ratio test clearly favored the second LMM. Next, we took the optimal random effects structure 2) and added fixed effects for model-based signatures in bilateral hippocampus, VS, and vmPFC, together with their respective two-way interactions with drinking intention. As model-based signatures across the three brain regions were correlated (Table S3), effects of each of the three predictors should be interpreted as the effect of the BOLD signal unique to that region and not co-varying with the BOLD signals in the two other regions, respectively.

We additionally computed complementary LMMs quantifying model-based behavior by the MB score, i.e. by individual beta estimates of the outcome × transition interaction from the logistic mixed-effects regression analysis of stay/switch behavior, which is independent of computational model assumptions. To test whether the predictive value of model-based behavior and its neural correlates goes beyond the one achieved by domain-general metrics of executive functioning, reasoning, and working memory, we repeated LMMs with additional *z*-standardized fixed effects for the number of correct digits on the DSST, the proportion of correct matrices on the Raven Standard Progressive Matrices, and the length of the maximum backwards remembered digit span on the DSBW as well as their respective interactions with drinking intention.

While LMMs are generally considered robust against violations of model assumptions [51], we re-estimated all LMMs using robust modeling [45, 52] to account for potential effects of observed assumption violations (Figure S2). Becauses the dependent variable contained a large number of zeros, resulting in a bimodal distribution, we additionally estimated GLMMs predicting the daily probability of drinking (gram alcohol/day > 0) vs. abstinence (gram alcohol/day = 0). GLMMs had the same fixed and random effects structure as LMMs. For standard (G)LMMs, we provide *p*-values based on the Wald degrees of freedom and two-sided 95% profile (for LMMs) or Wald (for GLMMs) confidence intervals for the fixed effects. For robust LMMs [45, 52], *p*-values were based on the Wald degrees of freedom of the corresponding standard LMM [53].

## Results

### Behavioral and neural correlates of model-based behavior in the two-step task

Conforming with previous studies [8, 32, 49, 50, 54], participants used a mixture of model-based and model-free behavior in the two-step task, evident in a medium level of computational parameter *ω* (*M* ± *SD* = 0.63 ± 0.22), as well as a main effect of outcome (*β* = 0.66, *z* = 11.17, 95% CI [0.56 0.77], *p* < 0.001) and an outcome x transition interaction (*β* = 1.35, *z* = 10.23, 95% CI [1.14 1.56], *p* < 0.001) when predicting stay/switch behavior in first-stage choices using logistic mixed-effects regression (Tables S4, S5). Simulation analyses using the hybrid reinforcement learning model showed acceptable parameter recovery for *ω* (*r* = 0.82, Supplementary Material, Table S6). In the EMA sample, AUD severity was not significantly associated with the degree of model-based vs. model-free behavior, captured in the computational parameter *ω* (‘moderate’ vs. ‘mild’: *β* = −0.01, *t*(64) = −0.08, 95% CI [−0.13 0.12], *p* = 0.934; ‘severe‘ vs. ‘mild’: *β* = 0.04, *t*(64) = 0.52, 95% CI [−0.11 0.18], *p* = 0.604; Figure S3B) or the MB score from the logistic mixed-effects regression analysis (‘moderate’ vs ‘mild’: *β* = −0.05, *t*(64) = −0.24, 95% CI [−0.50 0.39], *p* = 0.811; ‘severe’ vs. ‘mild’: *β* = 0.08, *t*(64) = 0.32, 95% CI [−0.42 0.59], *p* = 0.748). Moreover, model-based behavior was not associated with performance in the DSST ($\omega$: *ρ* = 0.06, *p* = 0.664; MB score: *ρ* = −0.01, *p* = 0.909), the Raven Standard Progressive Matrices ($\omega$: *ρ* = 0.21, *p* = 0.094; MB score: *ρ* = 0.17, *p* = 0.162), or the DSBW ($\omega$: $\rho$=0.13, *p* = 0.287; MB score: *ρ* = 0.19, *p* = 0.131).

**Fig. 2:**
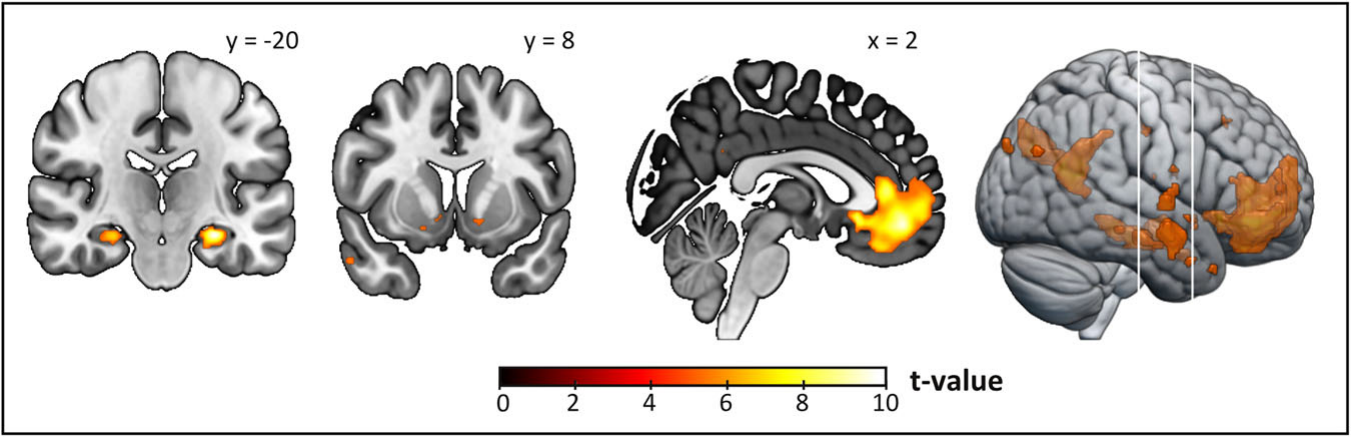
Neural correlates of model-based prediction error components. Blood oxygenation level dependent signal associated with unique model-based reward prediction error components according to Daw et al. [8] was evident in several cortical and subcortical regions, i.e., hippocampus, ventral striatum (VS), and ventromedial prefrontal cortex (vmPFC). Results are displayed at *p*_FWE_ ≤ 0.05 whole-brain corrected.

A whole-brain analysis of blood oxygenation level dependent (BOLD) responses correlating with unique model-based RPE components revealed a network comprising the bilateral VS (right: [x = 10, y = 10, z = −8], *Z* = 5.05, *p*_FWE_ = 0.018; left: [x = −12, y = 8, z = −8], *Z* = 5.24, *p*_FWE_ = 0.007), hippocampus (right: [x = 28, x = −18, z = −16], *Z* = 6.50, *p*_FWE_ < 0.001; left: [x = −28, y = −20, z = −16], *Z* > 8.00, *p*_FWE_ < 0.001), and ventromedial prefrontal cortex (vmPFC, right: [x = 2, y = 46, z = −10], *Z* > 8.00, *p*_FWE_ < 0.001; Fig. 2, Table S7) in participants performing the two-step task. These same regions also encoded model-free RPEs (Table S8). In accordance with the behavioral findings, model-based neural signatures did not differ significantly between AUD severity groups (no significant clusters at *p*_FWE_ <0.05 whole-brain corrected).

### Model-based behavior and related neural signals modulate the implementation of drinking intentions in AUD

Participants provided valid EMA data over a period of *M* ± *SD* = 272.21 ± 99.48 days. On average, they consumed 31.73 ± 47.57 g alcohol per day (23.96 ± 34.00 g for women, 34.46 ± 51.23 g for men). The intention to ‘drink less than usual’ was present on 24.90% of study days across participants and the individual proportion of days with this intention was not associated with the degree of model-based behavior ($\omega$: *ρ* = −0.05, *p* = 0.659, Figure S3E; MB score: *ρ* = −0.06, *p* = 0.615). For further descriptive EMA statistics, see Table S1 and Figure S3.

When regressing log daily alcohol intake on the parameter *ω* and drinking intention, we found a significant interaction between participants’ intention to ‘drink less than usual’ (contrast 1) and model-based behavior (*β* = −1.06, *t*(11562) = −3.31, 95% CI [−1.71 −0.42], *p* = 0.001; Table S9). Individuals with higher model-based behavior showed a lower daily alcohol consumption in weeks in which they had the intention to ‘drink less than usual’ compared to weeks in which they had ‘no specific intention’. For individuals with lower model-based behavior, the amount of daily alcohol intake was less affected by whether they intended to reduce their consumption or not. This moderation effect was also evident when quantifying model-based behavior by the MB score derived from the logistic mixed-effects regression of stay/switch behavior (Table S10) and when controlling for individual differences in executive functioning, reasoning, and working memory (Table S11).

**Fig. 3:**
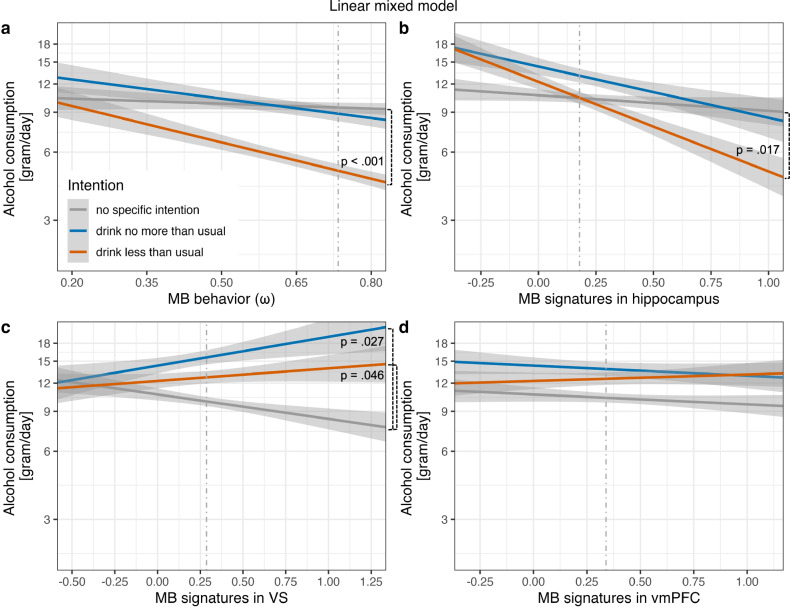
Partial effects for the four interactions including drinking intention from the linear mixed model predicting daily alcohol intake (Table 1). Individuals with **a** a higher degree of model-based behavior in the two-step task and **b** higher mean model-based signatures in bilateral hippocampus exhibited lower alcohol consumption on days on which they had the intention to ‘drink less than usual’ (vs. ‘no specific intention’). Individuals with **c** higher mean model-based signatures in bilateral ventral striatum (VS) showed higher alcohol consumption on days on which they had the intention to ‘drink less than usual’ or to ‘drink no more than usual’ (vs. ‘no specific intention’, respectively). Model-based signatures in **d** the ventromedial prefrontal cortex (vmPFC) did not interact with drinking intention. Values are plotted on a log-scale because log daily alcohol consumption was used as dependent variable in linear mixed models. Figure inlays in **b–d** display anatomical bilateral masks for regions of interest (hippocampus, VS, and vmPFC) used to extract mean blood oxygenation level dependent signal correlated with unique model-based reward prediction error components according to Daw et al. [8]. Grey shaded areas indicate 95% confidence intervals. Dashed grey vertical lines represent the median of the respective predictor variable.

When we added model-based neural signatures as regressors to the LMM predicting log daily alcohol intake, both model-based behavior (*β* = −1.06, *t*(11553) = −3.49, 95% CI [−1.68 −0.45], *p* < 0.001; Table 1, Fig. 3a) and model-based signatures unique to bilateral hippocampus (*β* = −0.75, *t*(11553) = −2.39, 95% CI [−1.38 −0.12], *p* = 0.017; Table 1, Fig. 3b), as well as unique to bilateral VS (*β* = 0.38, *t*(11553) = 2.00, 95% CI [0.01 0.76], *p* = 0.046; Table 1, Fig. 3c) interacted with participants’ intention to drink less. In weeks in which participants had the intention to reduce their drinking, individuals with more model-based behavior, stronger encoding of model-based RPE components in hippocampus, and weaker model-based signatures in VS exhibited lower daily alcohol consumption compared to weeks with no intention to reduce drinking. Specifically, at an average level of model-based signatures in hippocampus, VS, and vmPFC, the estimated marginal mean of daily alcohol intake on days with the intention to ‘drink less than usual’ was 5.89 g (SE = 3.37 g) lower for individuals with the highest (2.47 g [95% CI: 1.22–3.73 g]) compared to the lowest (8.36 g [95% CI: 2.37–14.35 g]) observed values in model-based behavior. When additionally considering significant neural predictors, an individual with the highest observed levels of model-based behavior and of hippocampal encoding of model-based RPEs as well as the weakest observed model-based VS signatures exhibited a 17.52 g (SE = 15.00) lower estimated marginal mean of alcohol consumption on days with a reduction intention (0.39 g [95% CI: −1.67–2.46 g]) compared to a person with the opposite combination of extreme predictor values (17.92 g [95% CI: −9.88–45.71 g]). Model-based signatures in bilateral VS additionally interacted with the intention to ‘drink no more than usual’ (*β* = 0.54, *t*(11553) = 2.21, 95% CI [0.06 1.06], *p* = 0.027; Table 1, Fig. 3c), with a 6.21 g (SE = 8.99 g) difference in marginal means on days with said intention between individuals with the highest (11.75 g [95% CI: −1.57–25.06 g]) and lowest (5.54 g [95% CI: 0.45–10.64 g]) observed model-based signatures in VS, all other predictors being at their average level.

**Table 1:** Linear mixed-effects regression model predicting daily alcohol consumption in *n* = 67 participants with alcohol use disorder by weekly drinking intentions, model-based behavior (*ω)* in the two-step task, and model-based neural signatures.

Fixed effect	Linear mixed model						Generalized linear mixed model					
	*β*	*SE*	95% CI		*t* (*df* = 11553)	*p*	*Odds Ratios*	*SE*	95% CI		*z*	*p*
			LL	UL					LL	UL		
Intercept	2.37	0.35	1.67	3.07	6.73	**<0.001**	1.94	0.82	-0.16	1.49	1.57	0.116
Drink no more than usual	0.29	0.28	−0.26	0.86	1.05	0.295	1.56	0.56	−0.26	0.15	1.23	0.218
Drink less than usual	0.14	0.23	−0.32	0.59	0.60	0.545	1.08	0.33	−0.52	0.68	0.26	0.794
*ω*	−0.17	0.49	−1.14	0.79	−0.35	0.727	0.70	0.40	−1.50	0.78	−0.62	0.535
MB signatures hippocampus	−0.16	0.51	−1.18	0.86	−0.31	0.757	1.01	0.61	−1.19	1.20	0.01	0.991
MB signatures VS	−0.25	0.31	−0.87	0.36	−0.81	0.420	0.72	0.27	−1.05	0.40	−0.89	0.375
MB signatures vmPFC	−0.10	0.34	−0.78	0.58	−0.30	0.767	0.76	0.31	−1.07	0.52	−0.68	0.495
Drink no more than usual × *ω*	−0.49	0.38	−1.26	0.29	−1.27	0.204	0.56	0.27	−1.54	0.37	−1.20	0.231
Drink less than usual × *ω*	−1.06	0.31	−1.68	−0.45	−3.49	**<0.001**	0.31	0.13	−1.98	−0.35	−2.81	**0.005**
Drink no more than usual × MB signatures hippocampus	−0.36	0.39	−1.15	0.41	−0.94	0.347	0.59	0.28	−1.46	0.42	−1.09	0.276
Drink less than usual × MB signatures hippocampus	−0.75	0.32	−1.38	−0.12	−2.39	**0.017**	0.43	0.18	−1.66	−0.01	−1.99	**0.047**
Drink no more than usual × MB signatures VS	0.54	0.25	0.06	1.06	2.21	**0.027**	1.87	0.57	0.03	1.23	2.05	**0.040**
Drink less than usual × MB signatures VS	0.38	0.19	0.01	0.76	2.00	**0.046**	1.65	0.41	0.01	0.99	2.002	**0.045**
Drink no more than usual × MB signatures vmPFC	−0.002	0.26	−0.54	0.51	−0.01	0.994	0.91	0.30	−0.73	0.55	−0.28	0.778
Drink less than usual × MB signatures vmPFC	0.17	0.22	−0.27	0.60	0.77	0.441	1.27	0.37	−0.33	0.81	0.83	0.407

‘Drink less than usual’ and ‘drink no more than usual’ refer to dummy-coded drinking intention, comparing log daily alcohol consumption (for linear mixed model) or probability of drinking (for logistic mixed-effects regression as special case of a generalized linear mixed-model) on days with this intention vs. on days with ‘no specific intention’ (reference level), respectively. Significant *p*-values are highlighted in bold. LL, lower limit; MB signatures, blood oxygenation level dependent signal associated with unique model-based reward prediction error components according to Daw et al. [8]; *SE*, standard error of the mean; UP, upper limit; vmPFC, ventromedial prefrontal cortex; VS, ventral striatum; *ω*, parameter quantifying the degree of model-based behavior in the two-step task; 95% CI: two-sided 95% profile (for linear mixed model) or Wald (for generalized linear mixed model) confidence intervals.

There were no significant main effects, indicating that neither model-based behavior nor its neural signatures predicted daily alcohol consumption when individuals had ‘no specific intention’ and providing no evidence for a direct effect of the intention to ‘drink less than usual’ on daily alcohol consumption when controlling for model-based behavior and its neural signatures. No further interactions were statistically significant (Table 1). Both the complementary analysis using the MB score derived from the logistic mixed-effects regression of stay/switch behavior instead of *ω* (Table S12) as well as the LMM including control regressors for measures of executive functioning, reasoning, and working memory confirmed all four significant interactions (Table S13).

**Fig. 4:**
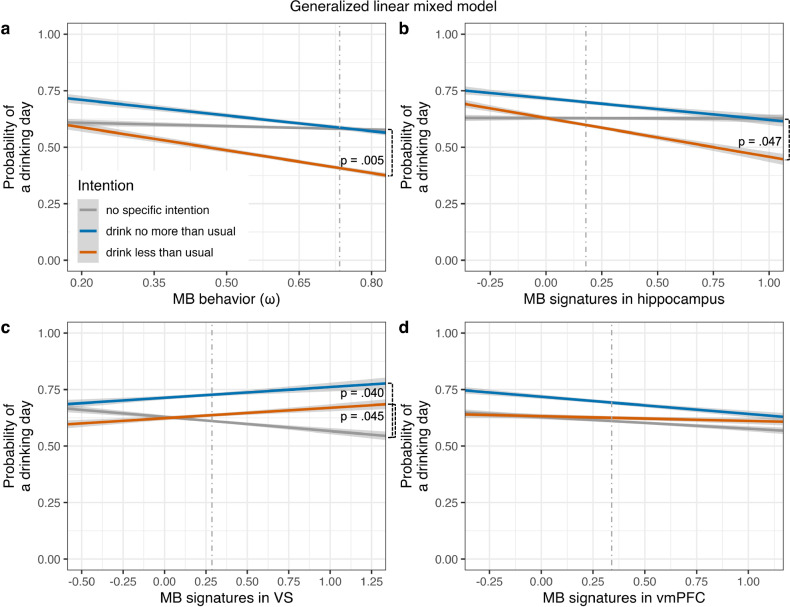
Partial effects for the four interactions including drinking intention from the generalized linear mixed model predicting the daily probability of drinking (Table 1). Individuals with **a** a higher degree of model-based behavior in the two-step task and **b** higher mean model-based signatures in bilateral hippocampus had a lower probability of drinking at all on days on which they had the intention to ‘drink less than usual’ (vs. ‘no specific intention’). Individuals with **c** higher mean model-based signatures in bilateral ventral striatum (VS) showed a higher probability of drinking at all on days on which they had the intention to ‘drink less than usual’ or to ‘drink no more than usual’ (vs. ‘no specific intention’, respectively). Model-based signatures in **d** the ventromedial prefrontal cortex (vmPFC) did not interact with drinking intention. Figure inlays in **b–d** display anatomical bilateral masks for regions of interest (hippocampus, VS, and vmPFC) used to extract mean blood oxygenation level dependent signal correlated with unique model-based reward prediction error components according to Daw et al. [8]. Grey shaded areas indicate 95% confidence intervals. Dashed grey vertical lines represent the median of the respective predictor variable.

Robust LMM re-estimation reproduced all significant effects (Tables S14–S19). Moreover, when predicting the probability of drinking (vs. abstinence) on a given day in GLMMs, we could confirm all results from LMMs using *ω* as a predictor (Fig. 4, Table 1, Table S20), also when controlling for individual differences in measures of cognitive functioning (Tables S21, 22; for GLMMs using MB score, see Tables S23, 24). As GLMMs disregard information on gram alcohol consumed on a drinking day, this suggests that the effects observed in LMMs are driven by intentional abstinence rather than controlled drinking.

## Discussion

In this study, higher model-based behavioral control, stronger hippocampal and weaker ventral striatal model-based signatures were associated with a stronger effect of intentions to limit drinking on daily alcohol intake in individuals with AUD. Our findings demonstrate the ecological and predictive validity of experimental conceptualizations of model-based behavior and highlight its potential role in exerting control over day-to-day alcohol consumption.

Most studies investigating the proposed gradual shift from goal-directed towards habitual behavior in AUD [3, 55] employed the two-step task [8, 32]. However, the majority did not observe group differences in model-free or model-based behavior between individuals with AUD or at-risk populations and healthy participants [27–30] (but see [56]). Similarly, there is little evidence for an association between AUD severity and the balance between model-free vs. model-based behavior in both high-risk samples [56] and the general population [57] (but see [50]). This is in line with our finding that the relative degree of model-based behavior and associated neural signatures are not associated with AUD severity per se. Instead, previous results suggest model-based behavior as a predictor for within-person long-term drinking outcomes, such as 12-month relapse status [28] or abstinence duration [29]. Likewise, young men with lower model-based behavior exhibited a steeper increase in alcohol consumed per binge drinking occasion over a 3-year follow-up period [31]. While these studies [28, 29, 31] relied on retrospective evaluation of alcohol consumption, we acquired high-temporal-resolution data using EMA. Compared with retrospective alcohol consumption reporting over long time periods, a prominent cross-validation study [33] showed that e-diary ratings capture more drinking days, a higher alcohol intake, a greater number of binge episodes, and a faster alcohol consumption rate, thereby increasing reliability of alcohol intake reports [34].

Using this methodology, we show that model-based behavior moderates the effect of intentions to limit consumption on alcohol intake in individuals with AUD. While Chen et al. [31] demonstrated that a higher degree of model-based behavior is a protective factor against developing binge-drinking, our results suggest that the effect of model-based control on drinking depends on the individual intention. Interestingly, individuals with a higher degree of model-based behavior did not set more reduction intentions, but if they did so, they were more successful at implementing them. Importantly, this effect was still present when controlling for individual differences in domain-general cognitive measures. Whether these findings are specific to individuals with AUD or generalize to other (at-risk) populations from the compulsive spectrum [29, 50] or the general population lies beyond the scope of the present study, as we did not include respective control groups.

On the neural level, previous studies have shown that model-free and model-based learning signals are encoded in partially overlapping brain regions, most notably the VS [8, 13, 28, 30] which has been implicated in reward learning [58]. Model-free learning is more strongly associated with activity in the basal ganglia, and model-based learning with prefrontal and limbic activity [13], including the hippocampus [14–17]. Neuroimaging studies using the two-step task speak against overall alterations in BOLD correlates of model-free or incremental model-based RPE components in AUD or at-risk populations [28, 30]. This is in line with the lack of evidence for an association between AUD severity and whole-brain model-based signatures in our study. However, dovetailing with behavioral findings, model-based and model-free neural correlates have been shown to predict drinking outcomes within participants. Specifically, weaker model-based neural signatures in medial prefrontal cortex differentiated individuals with AUD who experienced relapse during follow-up from those who remained abstinent and from healthy participants [28]. Moreover, stronger model-free RPE-associated VS and vmPFC activity was related to higher alcohol consumption over a 3-year follow-up period in young male social drinkers [31].

We show that stronger model-based hippocampal signatures enhance participants’ ability to implement their intention to limit drinking. Both Sebold et al. [28] and Chen et al. [31] focused on VS and vmPFC when predicting drinking outcomes, as hippocampal learning signals during the two-step task did not survive whole-brain correction [28, 30]. This contrasts with our results, where model-based signatures were strongest within the left hippocampus (Table S6). Stronger hippocampal engagement in model-based learning has been suggested to be induced by the task environment [13, 15]. Whereas the former studies [28, 31] used abstract geometric stimuli and no background story, our gamified two-step version and similar adaptations [15, 32, 57, 59] involve navigating between planets, with transitions being spatially related and embedded in a story-like narrative [13, 15]. Beyond its role in episodic memory and spatial navigation [60–62], both animal and human studies suggest that the hippocampus supports model-based decision-making [14–16, 63, 64]. Transient inactivation of bilateral dorsal hippocampus selectively impaired model-based planning in a two-step task for rodents [16]. This region, together with cortical areas, provides a representation of task contingencies used for goal-directed choices [63, 64]. Conceptual similarities between allocentric spatial memory and forward planning during goal-directed decision-making [15, 16] are supported by the finding that both model-based behavior in the two-step task and spatial memory are hippocampus-dependent and impaired in epilepsy patients with unilateral hippocampal resection [15]. It has been further suggested that hippocampal replay, i.e., hippocampal place cell activity representing temporally compressed versions of state trajectories during awake rest or sleep, is a neural substrate enabling goal-directed behavior [65–67]. Future studies should further examine the role of the hippocampus in model-based decision-making in AUD.

Moreover, participants with stronger model-based signatures in the VS consumed more alcohol in weeks in which they intended to ‘drink less than usual’ or to ‘drink no more than usual’ compared to weeks with ‘no specific intention’, respectively. These interactions were unexpected, especially given a direction opposite to the influence of model-based behavior and hippocampal signatures in implementing the intention to limit drinking. One possible explanation is that distributed model-based neural signatures reflect distinct computational building blocks of model-based behavior, not model-based behavior per se [68]. However, the interactions involving model-based VS signatures must be interpreted with caution because both are computed relative to a numerically negative slope of model-based signatures in the VS in weeks with ‘no specific intention’, that is, the base of the dummy contrast. Moreover, an interpretation for the interaction between model-based VS signatures and the intention to drink ‘no more than usual’ is hardly possible because, like having ‘no specific intention’, the successful implementation of this intention does not imply a within-subject change in consumption. Accordingly, model-based behavior did not interact with the intention to drink ‘no more than usual’. Future studies should aim to further dissociate the functional roles of VS and hippocampus in model-based behavior and their respective relevance for AUD.

Several aspects of the present study merit discussion. First, the suitability of the two-step task for investigating habitual behavior has been criticized, as the computational formalization of model-free learning contrasts with traditional definitions of habits as outcome-independent stimulus-response associations [69]. Therefore, our study focused on the role of goal-directed, i.e., model-based, behavioral control. Future research should employ paradigms that might be better suited to capture habitual behavior, such as outcome devaluation, contingency degradation, or action sequence learning [11, 70], and assess its relation to real-life consumption behavior. Furthermore, the ability to clearly dissociate model-based from model-free learning in this task has been questioned, as seemingly model-free behavior can result from poorly understood task instructions [71]. Conversely, simulation studies indicate that, under specific conditions, model-free agents appear model-based [72]. We attempted to minimize these issues by employing a gamified task [32] with instructions conveying a detailed storyline [71], as well as a correction to logistic mixed-effects regression analyses of two-step data as previously suggested [49, 72]. Still, the fact that strategies beyond model-free and model-based learning can be used during the two-step task [22, 71, 72] remains a limitation of current computational models and thus of our study. Future task designs should allow differentiation between reinforcement learning strategies on a continuum between model-free and model-based control, such as successor representation algorithms [12, 73, 74]. These might provide a more accurate computational representation of neuronal activity [14]. Moreover, while model-based behavior and associated neural signatures were unrelated to AUD severity per se in our sample of mainly mild-to-moderate cases, future studies with larger sample-sizes could investigate how disorder severity or other contextual factors like negative mood states [26] or stress [75] affect the relation between model-based behavior and intentional reduction of alcohol consumption in AUD. In this context, it is important to consider model-based control not only as a stable trait but also as a dynamic, situationally modifiable process. Recent findings suggest that incentives can enhance model-based control even in individuals with elevated symptom levels [57], highlighting its potential as a flexible treatment target in real-world settings. Lastly, our LMM analysis does not allow to elucidate the dynamic temporal relationships between intentions and alcohol consumption over time, as we average across days with a specific intention. We encourage to explore the applicability of, for example, multi-level vector auto-regression or continuous-time models to our data to answer different, albeit interesting research questions [76].

In conclusion, we demonstrate the ecological validity and predictive power of behavioral and possibly also of neural markers of model-based behavior beyond domain-general cognitive performance in the context of control over alcohol intake in day-to-day life in AUD. While our study design supports the notion of temporal directionality (i.e., model-based behavior was assessed at the beginning of the EMA period and drinking intentions were recorded prospectively), it was of observational nature. To proceed towards causal conclusions, future studies could implement repeated assessments of model-based behavior or even experimental manipulations in everyday life [77, 78]. This could ultimately reinforce what our current findings hint at: that goal-directed behavior is a promising target for behavioral interventions to establish control over alcohol intake. Under this premise, AUD participants could benefit from a personalized treatment approach considering their degree of goal-directed behavior. While the economic viability of assessing model-based neural signatures is harder to establish, behavioral assessments such as the two-step task seem feasible in clinical settings. Specifically, individuals with higher levels of model-based behavior should best benefit from cognitive interventions helping them set and maintain realistic and manageable drinking intentions. For individuals displaying less model-based behavior, however, these interventions might have limited effectiveness. Instead, treatments focusing on maladaptive learning processes (e.g., virtual reality cue-exposure or reconsolidation-based approaches) should be beneficial and could be complemented by interventions aiming to increase goal-directed behavior (for a review of such interventions, see [79]).

## Supplementary information

Supplementary Material

## Data Availability

Code is publicly accessible at github.com/agschlagenhauf/MB_EMA.git. Data are available upon request from the corresponding authors.
