# Supplementary material for: Goal-directed behavior and hippocampal activity predict real-life impact of drinking intentions in alcohol use disorder
Source: Transl Psychiatry. 2025 Oct 20;15:425. doi: 10.1038/s41398-025-03660-5 (PMC12537898; doi:10.1038/s41398-025-03660-5)
Supplement: Supplementary file 1 — Supplementary Material [file 41398_2025_3660_MOESM1_ESM.docx]

Supplementary Material for

**Goal-directed behavior and hippocampal activity predict real-life impact of drinking intentions in alcohol use disorder**

Claudia Ebrahimi, PhD_1_*^1,2^ & Milena P. M. Musial, M.Sc._1_*^1,3,4,5^, Nuria Doñamayor, PhD^1^, Diana S. Prychynenko, PhD^1,3^, Erik L. Bode, M.Sc.^1,3,4^, Rainer Spanagel, PhD^6,7^, Andreas Heinz, MD, PhD^7,8,9^, Lorenz Deserno, MD^9,10^, Michael N. Smolka, MD^9^, Ulrich Ebner-Priemer, PhD^7,11,12^, Reinhold Kliegl, PhD^13^, Tanja Endrass, PhD_2_^2^, Markus Reichert, PhD_2_^11,12,14^, Michael Rapp, MD, PhD_2_^7,13^, and Florian Schlagenhauf, MD_2_^1,4,5^

^1^Charité – Universitätsmedizin Berlin, corporate member of Freie Universität Berlin and Humboldt-Universität zu Berlin, Department of Psychiatry and Neurosciences | CCM, NeuroCure Clinical Research Center, 10117 Berlin, Germany

^2^Institute of Clinical Psychology and Psychotherapy, Faculty of Psychology, Technische Universität Dresden, 01062 Dresden, Germany

^3^Humboldt-Universität zu Berlin, Faculty of Life Sciences, Department of Psychology, 10099 Berlin, Germany

^4^Charité – Universitätsmedizin Berlin, corporate member of Freie Universität Berlin and Humboldt-Universität zu Berlin, Einstein Center for Neurosciences Berlin, 10117 Berlin, Germany

^5^Charité – Universitätsmedizin Berlin, corporate member of Freie Universität Berlin and Humboldt-Universität zu Berlin, Bernstein Center for Computational Neuroscience, 10117 Berlin, Germany

^6^Institute of Psychopharmacology, Central Institute of Mental Health, Medical Faculty Mannheim, University of Heidelberg, 68159 Mannheim, Germany

^7^German Center for Mental Health (DZPG), Germany

^8^Department of Psychiatry and Psychotherapy, Universität Tübingen, 72076 Tübingen, Germany

^9^Department of Psychiatry and Psychotherapy, Technische Universität Dresden, 01062 Dresden, Germany

^10^Department of Child and Adolescent Psychiatry, Psychosomatics and Psychotherapy, University Hospital Würzburg, 97080 Würzburg, Germany

^11^Mental mHealth Lab, Department of Applied Psychology, Institute of Sports and Sports Science, Karlsruhe Institute of Technology, 76131 Karlsruhe, Germany

^12^Department of Psychiatry and Psychotherapy, Central Institute of Mental Health, Medical Faculty Mannheim, University of Heidelberg, 68159 Mannheim, Germany

^13^Social and Preventive Medicine, University of Potsdam, 14476 Potsdam, Germany

^14^Department of Sport and Exercise Science, Faculty of Natural and Life Sciences, University Salzburg, 5020 Salzburg, Austria

_1_These first authors contributed equally

_2_These senior authors contributed equally

*Corresponding authors: Claudia Ebrahimi, Email: [claudia.ebrahimi@charite.de](mailto:claudia.ebrahimi@charite.de); Milena P. M. Musial, Email: milena.musial@charite.de

**This PDF file includes:**

Supplementary Text

Supplementary Tables

Supplementary Figures

Supplementary References

**Other supporting materials for this manuscript include the following:**

Code at [github.com/agschlagenhauf/MB_EMA.git](https://github.com/agschlagenhauf/MB_EMA.git)

Exclusion criteria

This study was part of the Collaborative Research Center 265 – Regaining and Losing Control over Drug Intake (ReCode) ^1^. Participants were recruited from the general population via public advertisement at Charité – Universitätsmedizin Berlin and Technical University Dresden. Some of the advertisements targeted individuals who reported regular alcohol use and alcohol-related problems, such as impaired control over consumption. AUD criteria were assessed on-site using the Structure Clinical Interview ^2^ for the Diagnostic and Statistical Manual of Mental Disorders (DSM)-5) ^3^. Pre-established inclusion criteria were the ability to provide full informed consent, to use self-assessment scales, and to understand the study protocol, as well as sufficient knowledge of the German language. The following pre-established exclusion criteria were applied: lifetime history of bipolar I and psychotic disorder, current (past 12 months) substance use disorder other than alcohol, nicotine or cannabis use disorder, according to the DSM-5 ^3^; current DSM-5 ^3^ manic episode or severe major depressive episode or acute suicidality, borderline personality disorder, consumption of alcohol and psychoactive substances other than tobacco and cannabis within 24 hours prior to each assessment, verified by drug urine testing for amphetamines, cocaine, and opioids, and by breath alcohol testing allowing to estimate blood alcohol level (max. 0.0% on the day of assessment); explicit desire for alcohol abstinence; clinical indication for medically supervised alcohol detoxification or acute therapeutic intervention; history of qualified inpatient detoxification; current addiction-related treatment (inpatient, outpatient, day clinic, or long-term therapy); history of severe traumatic brain injury or other severe central neurological disorder (e.g. dementia, Parkinson's disease, multiple sclerosis, epilepsy); pregnancy or breastfeeding; metal implants or other MRI contraindications.

Statistical power considerations

Power analyses for linear mixed models (LMM) or generalized linear mixed models (GLMM) require a priori effect sizes not only for fixed effects and their interactions, but also for the size of variance components and correlation parameters. Unfortunately, for this study, there was no knowledge about these model parameters or even which of them would be supported during model selection. Therefore, we did not conduct an a priori power analyses.

Two-step task

The two-step task adapted from Daw et al. ^4^ and Kool et al. ^5^ was presented in Pychtoolbox version 3.0.16 ^6–8^ for MATLAB 2019b (The MathWorks, Inc., Natick, Massachusetts, United States). Before completing the task during functional magnetic resonance imaging (fMRI), all participants received detailed instructions and performed a 20-trial training version outside the scanner to familiarize themselves with the task structure. The task consisted of 201 trials, each of which started on planet earth (first-stage state; duration: 2 s + jittered time period [M = 1.49 ± 0.28 s, range 1-2 s]), where participants had to choose one of two spaceships. Each spaceship (first-state action) led to one extraterrestrial planet (second-stage state) with probability P = .7 (common transition) and to the other extraterrestrial planet with P = .3 (rare transition). Trial order was pseudorandomized, so that rare transitions could occur in no more than three consecutive trials. At each extraterrestrial planet (duration: 2 s + jittered time period [M = 1.49 ± 0.28 s, range 1-2 s]), participants saw two aliens in the corresponding color. Participants had to choose one of the aliens to receive the comet gold (reward) or anti-matter (punishment) they had mined. The outcomes (1.5 s) associated to each alien (second-stage action) changed continuously and independently over trials according to Gaussian random walks with a drift rate of σ = 0.1, reflecting boundaries at –5 (five pieces of anti-matter) and +5 (five pieces of comet gold), and no 0 point option. Participants were informed that, at the end of the task, one third of the trials would be randomly selected and the points obtained translated into Euro (1 point = 0.1 €). Not known to participants, we defined minimum and maximum rewards of 5 € and 10 €, respectively. The position (left/right) of the first- and second-stage stimuli was randomized across trials. Trials were aborted if no or an invalid key was pressed during stages 1 or 2. Trials were separated by a jittered inter-trial interval during which a fixation cross was shown (duration: 1 s + jittered time period [M = 1.03 ± 1.04 s, range 0.01-6 s]).


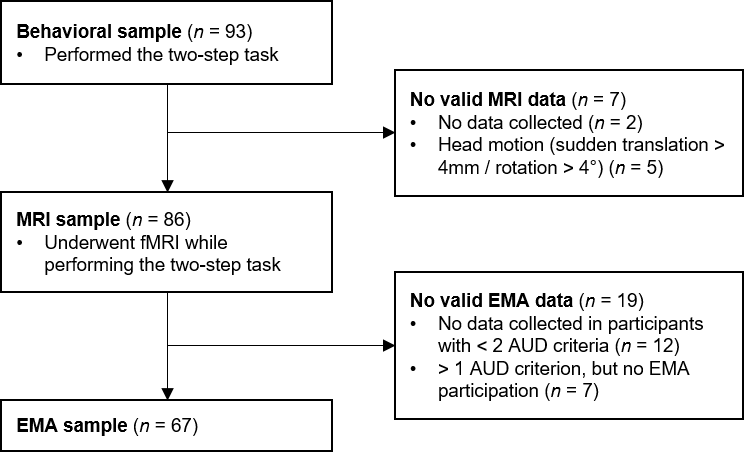


**Figure S1.** Participant flowchart. AUD, alcohol use disorder according to the Diagnostic and Statistical manual of Mental Disorders (DSM)-5 ^3^; EMA, ecological momentary assessment; (f)MRI, (functional) magnetic resonance imaging.

**Table S1.** Sample characteristics

| **Characteristic** | **Behavioral sample**  **(n = 93)** | **EMA sample**  **(n = 67)** |
| --- | --- | --- |
| Recruitment site (Berlin / Dresden; *n*) | 87 / 6 | 61 / 6 |
| Gender (female / male / other; *n*) | 34 / 59 / 0 | 20 / 47 / 0 |
| **At initial assessment** |  |  |
| Age (*M* ± *SD*; years) | 33.72 ± 9.11 | 35.18 ± 9.43 |
| School-leaving qualification (current student at high-school / school leaving certificate after grade 10 / technical college or higher education entrance qualification; *n*) | 1 / 13 / 76 ^a^ | 0 / 9 / 58 |
| AUD criteria past 12 months (*M* ± *SD,* range) | 3.76 ± 1.92, 0 – 8 | 4.30 ± 1.51, 2 – 7 |
| AUD severity (no AUD / mild / moderate / severe, *n*) | 13 / 27 / 34 / 19 | 0 / 21 / 29 / 17 |
| AUDIT score (*M* ± *SD*) | 13.97 ± 5.59 ^a^ | 15.42 ± 4.87 |
| Alcohol / day during past 3 months, as indicated in Quantity Frequency Questionnaire (*M* ± *SD*; standard drinks) | 2.69 ± 2.01 | 3.06 ± 2.04 |
| Current smoking (yes / no; *n*) | 38 / 55 | 26 / 41 |
| Smoking lifetime (yes / no; *n*) | 54 / 36 ^a^ | 42 / 23 ^d^ |
| Days smoked tobacco during past 3 months (*M* ± *SD*) | 31.79 ± 40.50 ^b^ | 32.81 ± 41.67 ^e^ |
| CUD criteria past 12 months (*M* ± *SD*) | 0.52 ± 1.63 | 0.72 ± 1.88 |
| Days smoked cannabis during past 3 months (*M* ± *SD*) | 6.63 ± 20.18 ^c^ | 8.92 ± 23.12 ^f^ |
| Correct digits on Digit Symbol Substitution Test ^9^ (*M* ± *SD*) | - | 71.70 ± 16.98 ^g^ |
| Proportion of correct matrices in Raven Standard Progressive Matrices ^10^ (*M* ± *SD*) | - | 0.57 ± 0.20 |
| Maximum backwards remembered digit span on Digit Span Backwards Task ^11^ (*M* ± *SD*) | - | 5.18 ± 1.20 ^h^ |
| **Ecological Momentary Assessment during 12-months period** |  |  |
| Alcohol consumption per day (*M* ± *SD*; gram) | - | 31.73 ± 47.57 |
| female |  | 23.96 ± 34.00 |
| male |  | 34.46 ± 51.23 |
| Drinking intention frequency (drink less than usual / drink no more than usual / no specific intention; %) | - | 24.90 / 23.42 / 51.68 |
| Days between first and last complete EMA (*M* ± *SD*) | - | 272.21 ± 99.48 |
| Days with complete EMA (*M* ± *SD*) | - | 172.72 ± 104.62 |
| Percent days with complete EMA out of days between first and last complete EMA per participant (*M* ± *SD*) | - | 61.37 ± 25.38 |

*Notes.* EMA statistics are based on valid data for a total of 11572 days across all participants. Drinking intention frequency refers to percentage of days across participants, whereby intention was assessed every 8 days and assigned to the days pertaining the respective intention. AUD, Alcohol Use Disorder according to the Diagnostic and Statistical manual of Mental Disorders (DSM)-5 ^3^; AUDIT, Alcohol Use Disorder Identification Test ^12^; CUD, DSM-5 Cannabis Use Disorder

^a^ based on *n* = 90; ^b^ based on *n* = 86; ^c^ based on *n* = 80; ^d^ based on *n* = 65; ^e^ based on *n* = 63;

^f^ based on *n* = 59; ^g^ based on *n* = 64, ^h^ based on *n* = 66

Ecological momentary assessment (EMA) acquisition details

To assess everyday alcohol consumption, we asked participants to install the movisensXS app (movisens GmbH, Karlsruhe, Germany) including an electronic diary (e-diary) on their smartphone. We provided participants with smartphones (Nokia 3.4, Nokia 6.2, and Nokia 7.2, Nokia, Espoo, Finland) for research purposes in case their own smartphones were incompatible with the app used in the study. Participants were prompted to complete an e-diary entry via the app once every second day. An acoustic, vibration and visual alarm was triggered at 12 PM but could be postponed up to 8 hours until 8 PM (in 5-minute to 8-hour intervals). If the alarm was ignored, it recurred five more times in 90-minute intervals, and a button to start the e-diary remained on the home screen of the app throughout the day to enable participants to start the questionnaire individually. Expense allowance was provided as a bonus payment for participants who reached over 80% compliance—calculated based on completed entries and missed alarms—with 20 € awarded after the first and second follow-ups, and 30 € after the third, for a maximum total of 70 € over the one-year assessment period.

Alcohol consumption and drinking intention were assessed via the following items in German language: “Denken Sie an gestern (think about yesterday) [second item: Denken Sie an vorgestern (think about the day before yesterday)]: Welche und wie viele alkoholische Getränke haben Sie konsumiert? (which and how many alcoholic drinks did you consume?)” and “Planen Sie, Ihren Alkoholkonsum die nächsten 8 Tage zu begrenzen? (do you plan to restrict your alcohol consumption in the next 8 days?)”, with 1 indicating "Nein, ich habe keine speziellen Vorsätze”, 2 indicating "Ja, ich möchte nicht mehr als üblich trinken”, and 3 indicating “Ja, ich möchte weniger als üblich trinken”.

**Table S2.** List of alcoholic drinks used in ecological momentary assessment

| **Drink type** | **Volume in liter** | **ABV^1^** |
| --- | --- | --- |
| No alcoholic drink |  |  |
| Small beer | 0.2 | 5.0% |
| Regular beer | 0.33 | 5.0% |
| Large beer | 0.5 | 5.0% |
| Small white wine | 0.1 | 11.0% |
| Regular white wine | 0.2 | 11.0% |
| Bottle of white wine | 0.75 | 11.0% |
| Small red wine | 0.1 | 12.0% |
| Regular red wine | 0.2 | 12.0% |
| Bottle of red wine | 0.75 | 12.0% |
| Sparkling wine | 0.1 | 11.5% |
| Bottle of sparkling wine | 0.75 | 11.5% |
| Fortified wine (e.g., port, Sherry) | 0.05 | 19.7% |
| Small liqueur (e.g., Contreau, Sambuca, Underberg, Jägermeister) | 0.02 | 20% |
| Large liqueur (e.g., Contreau, Sambuca, Underberg, Jägermeister) | 0.05 | 20% |
| Sweet liqueur (e.g., Aperol, Amaretto, Advocaat, Baileys, Batida de Coco) | 0.02 | 17% |
| Small spirit (e.g., fruit liqueur, cherry liqueur, Vodka, Gin, juniper, corn, brandy) | 0.02 | 40% |
| Large spirit (e.g., fruit liqueur, cherry liqueur, Vodka, Gin, juniper, corn, brandy) | 0.05 | 40% |
| Spirit (e.g., fruit liqueur, cherry liqueur, Vodka, Gin, juniper, corn, brandy) | 0.1 | 40% |
| Small spirit (strong) | 0.02 | 65% |
| Brandy (e.g., Brandy, Cognac, Armagnac, Metaxa) | 0.02 | 36% |

*Notes.* ^1^ABV = Alcohol by volume

Computational modeling of behavioral data

The task consisted of three states ($s_{A},s_{B},s_{C}$; Figure 1) across two stages. The first-stage state in trial $t$ will be denoted as $s_{1,t}$ and always equals $s_{A}$. Second-stage states in trial $t$ will be denoted as $s_{2,t}$ and are either $s_{B}$ or $s_{C}$.

We fitted sequential decision-making data from all participants (n=93) using the hybrid reinforcement learning model according to Daw et al. ^4^. As a model-free algorithm, we used SARSA(λ) temporal difference learning ^13^. Here, the model-free value $Q_{MF}$ of action $a$ in state $s$ at stage $i$ is updated in each trial $t$ according to

$Q_{MF}\left( s_{i,t},a_{i,t} \right)= Q_{MF}\left( s_{i,t-1},a_{i,t-1} \right)+\alpha_{i}\delta_{i,t}+\alpha_{i}{\lambda\delta}_{i+1,t}$ [1]

, where the prediction error $\delta_{i,t}$ is computed as

$\delta_{i,t}= r_{i,t}+Q_{MF}\left( s_{i+1,t-1},a_{i+1,t-1} \right)-Q_{MF}\left( s_{i,t-1},a_{i,t-1} \right)$ [2]

and is weighted by a stage-specific learning rate $\alpha_{i}$. The reinforcement eligibility parameter $\lambda$ indicates to what extent first-stage $Q$-values are updated by the final outcome in addition to the second-stage $Q$-value. As in Daw et al. ^4^, $\lambda$ is replaced between trials. As first-stage actions are not followed by immediate reward ($r_{1,t}$ = 0) and our task did not include third-stage states and actions ($Q\left( s_{2+1,t},a_{2+1,t} \right)$ = 0), reward prediction errors at the first and second stage are respectively computed as follows:

$\delta_{1,t}=Q_{MF}\left( s_{1+1,t-1},a_{1+1,t-1} \right)-Q_{MF}\left( s_{1,t-1},a_{1,t-1} \right)$ [3]

$\delta_{2,t}= r_{2,t}-Q_{MF}\left( s_{2,t-1},a_{2,t-1} \right)$ [4]

As in Daw et al. ^4^, we assumed that participants simply add up the encountered transitions of $s_{A},a_{A}\to s_{B}$ and $s_{A},a_{B}\to s_{C}$ and compare them to the sum of the encountered transitions $s_{A},a_{A}\to s_{C}$ and $s_{A},a_{B}\to s_{B}$ to decide between two predetermined transition structures: $P\left( s_{B} | s_{A},a_{A} \right)$ = 0.7 and $P\left( s_{C} | s_{A},a_{B} \right)$ = 0.7, or $P\left( s_{B} | s_{A},a_{A} \right)$ = 0.3 and $P\left( s_{C} | s_{A},a_{B} \right)$ = 0.3 ^4^.

The model-based $Q$-value of a first-stage state-action pair is then computed for each trial according to Bellman’s equation ^4,13^:

$Q_{MB}\left( s_{A},a_{j} \right)= P\left( s_{B} | s_{A},a_{j} \right) \max_{a\in(a_{A},a_{B})} Q_{MF}\left( s_{B},a \right)+ P\left( s_{C} | s_{A},a_{j} \right) \max_{a\in(a_{A},a_{B})} Q_{MF}\left( s_{C},a \right)$ [5]

At the second stage, both model-based and model-free $Q$-values are identical, $Q_{MB}\left( s_{2},a \right)$ = $Q_{MF}\left( s_{2},a \right)$.

Model-free and model-based $Q$-values are then combined $(Q_{comb}$). At the first stage, their relative weight is captured by the parameter *ω*:

$Q_{comb}\left( s_{1,t},a_{1,t} \right)={\omega Q}_{MB}\left( s_{1,t},a_{1,t} \right)+{(1-\omega)Q}_{MF}\left( s_{1,t},a_{1,t} \right)$ [6]

Pure model-free learning is indicated by *ω* = 0, while pure model-based learning is indicated by *ω* = 1.

To translate values of state-action pairs into choice probabilities, we used a softmax choice rule ^4^:

$P\left( a | s_{i,t} \right)=\frac{exp(\beta_{i}\left[ Q_{comb}\left( s_{i,t},a \right)+pi*rep\left( a \right) \right])}{\sum_{a'} exp(\beta_{i}\left[ Q_{comb}\left( s_{i,t},a' \right)+pi*rep\left( a' \right) \right])}$ [7]

, where $\beta$ is the inverse temperature parameter per stage $i$, indicating the degree of choice stochasticity. The free parameter $pi$ and the term $rep$ are included to capture repetition ($rep$ = 1) or switching ($rep$ = 0) ^14^ of first-stage choices (for further information, see Daw et al. ^4^).

Following Daw et al. ^4^, for fMRI analyses, we defined a generalized reward prediction error (RPE) equation taking into account combined model-free and model-based $Q$-values:

$\delta_{comb,i,t}= r_{i,t}+Q_{comb}\left( s_{i+1,t-1},a_{i+1,t-1} \right)-Q_{comb}\left( s_{i,t-1},a_{i,t-1} \right)$ [8]

The regressor indicating the difference between RPEs computed for *ω* = 1 and *ω* = 0 equals the partial derivative of the generalized RPE with respect to *ω*. To compute individual RPE trajectories, we used individual estimates for $\beta_{1},\beta_{2}, \alpha_{1}, \alpha_{2}, \lambda$, and $pi$.

Model estimation and model comparison

The seven free parameters of the reinforcement learning model $(\beta_{1},\beta_{2}, \alpha_{1}, \alpha_{2}, \lambda, pi, \omega)$ were estimated using hierarchical Bayesian analysis as implemented in the Stan software ^15^ using the RStan package ^16^ in R version 4.2.1 ^17^. Hierarchical Bayesian analysis estimates group-level parameters while taking into account individual differences: individual parameters are drawn from normal group-level parameter distributions, leading to stable and reliable parameter estimates ^18,19^. We used weakly informative ^19^ normal distributions (*M* = 0, *SD* = 1) as priors for group-level means and standard deviations, whereby the group-level standard deviation was constrained to a lower bound of zero.

Posterior distributions for the group-level parameters were sampled using the Hamiltonian Monte Carlo algorithm as implemented in Stan, with four chains and 10,000 iterations each, 500 of which were warmup iterations. $\hat{R}$-values were less than 1.1, indicating chains converged ^20^. The unconstrained individual parameter estimates of $\alpha_{1}, \alpha_{2}, \lambda, \omega$ were transformed to a range between 0 and 1, while the unconstrained individual parameter estimates of $\beta_{1},\beta_{2}$ were transformed to a range between 0 and ∞.

To further evaluate the performance of the hybrid reinforcement learning model, we additionally estimated a pure model-free reinforcement learning (RL) model (with free parameters $\alpha, \beta, \lambda, pi$) and the logistic mixed-effects regression on the stay probability in pairs of consecutive trials (with free parameters $outcome, transition, outcome*transition$ and $correct$*,* a predictor for correct choices) in Stan. To identify which model best explains first-stage choices on the 2-step task, the expected log pointwise predictive density (ELPD) as a measure for predictive accuracy was estimated using Pareto-smoothed importance sampling ^21^. To extract leave-one-out cross-validation estimates, subsampling (100 trials per participant) was used. A smaller ELPD (absolute) value suggests a better model fit to the data.

The pairwise comparison between the hybrid reinforcement learning model and model-free RL model revealed an estimated difference in ELPD of -185 (SE=75, 95% CI [-331, -39]) and of -673 (SE=84, 95% CI [-837, -509]) between the hybrid reinforcement learning model and the logistic regression model, favoring the hybrid model, respectively.

Mixed-effects logistic regression analysis of stay/switch behavior

In addition to computational modeling, we performed a mixed-effects logistic regression analysis on the behavioral data (see ^4,22–25^). Participants’ tendency to repeat (stay = 1) or change (switch = 0) the first-stage action in the preceding trial was predicted by outcome (effect-coded as reward = 0.5 and punishment = -0.5), transition type (common = 0.5, rare = -0.5), and their interaction. Additionally, as suggested by Smittenaar et al. ^24^ and Akam et al. ^25^, we added a predictor for correct choices, defined as the action with a common transition leading to the second-stage state with the currently highest reward probability (correct = 0.5, incorrect = -0.5) in order to capture variance otherwise shown to lead to overestimation of the outcome × transition interaction, i.e. of model-based influences on choice behavior ^24,25^. We used the full random effects structure, modeling individual intercepts and all predictors as random effects. In this analysis, the main effect of outcome is considered indicative of model-free behavior, while the outcome × transition interaction indicates model-based behavior ^4,5^. Individual beta estimates of the outcome × transition interaction (i.e. MB scores) were extracted for further analyses in the EMA sample.

FMRI data collection, preprocessing, first-level analyses, and mask creation

Functional and structural MRI was performed at identical 3-Tesla scanners (Trio, Siemens AG, Erlangen, Germany; 1 scanner per site) using a 64-channel head coil. Functional images were acquired using a T2*-weighted multiband echo-planar imaging (EPI) sequence (TR = 869 ms, TE = 38 ms, flip angle = 58°, matrix = 88*88, FOW = 210 mm, 60 slices, voxel size = 2.4 x 2.4 x 2.4 mm, multiband factor = 6). As anatomical reference, we acquired a T1-weighted structural image (TR = 2 s, TE = 2.01 ms, flip angle = 8°, matrix = 256*256, FOW = 256 mm, 208 slices, voxel size = 1 x 1 x 1 mm) as well as a dual gradient echo fieldmap to allow spatial correction of the EPI images.

FMRI preprocessing included slice time correction, realignment to the mean echo-planar imaging (EPI) volume, distortion correction using the acquired fieldmaps (unwarping), segmentation of the T1-weighted structural image, co-registration of the mean EPI to the segmented structural image, spatial normalization to Montreal Neurological Institute (MNI) space (2-mm isotropic voxel resolution), and smoothing using a 6-mm full-width at half-maximum Gaussian Kernel. Before performing first-level statistics, data were high-pass filtered using a filter of 128 s width.

First-level analyses were performed as previously described ^4^ and included a combined onset regressor for second-stage and reward, modeled as stick function, which was parametrically modulated by (i) model-free RPEs and (ii) unique model-based RPE components, i.e., the difference between model-based and model-free RPEs. Thereby, individual parametric modulator trajectories for (i) equal the model-free RPE (*ω* = 0) and for (ii) equal the difference between RPEs computed for *ω* = 1 and *ω* = 0 (see equation [8]). As the parametric modulator (ii) is zero at the time of reward onset, where model-free and model-based learning algorithms converge, difference values at second-stage onset were mean-centered within-subject to account for potential systematic BOLD differences between stages. The first-level model included two additional nuisance regressors: reward and first-stage onsets, both modeled as stick functions, with two parametric modulators at first-stage onset, i.e., the choice probability for first-stage action as given by equation [7], and its partial derivative with respect to *ω*. All onset regressors were convolved with the canonical HRF and its temporal derivative.

To account for variance related to head-motion, we adopted a 24-parameter de-noising strategy ^26^ including the six standard head-motion parameters, their first temporal derivatives and all quadratic terms as additional nuisance regressors. All onset regressors were convolved with the canonical HRF and its temporal derivative. For the bilateral ventral striatum (VS), the ventromedial prefrontal cortex (vmPFC), and the bilateral hippocampus, masks including voxels coding model-free and model-based RPEs were created using conjunction *t*-maps at *p* <.001 uncorrected. In order to ensure regional specificity, clusters were constrained to voxels within hippocampus and basal ganglia (for the VS), based on the WFU PickAtlas (http://www.fmri.wfubmc.edu/download.htm). For the vmPFC, a 10-mm sphere centered on the peak voxel in the conjunction analysis [x = 2, y= 46, z= −10] was used to generate the respective mask (see Figure 3).

**Table S3.** Intercorrelations of predictor variables for linear mixed-effects regression models in *n*=67 participants with alcohol use disorder

|  | **ω** | **MB score** | **MB signatures vmPFC** | **MB signatures hippo-campus** | **MB signatures VS** |
| --- | --- | --- | --- | --- | --- |
| ω | 1.00 | **.72** | .12 | -.01 | -.07 |
| MB score |  | 1.00 | .09 | -.05 | -.01 |
| MB signatures vmPFC |  |  | 1.00 | **.65** | **.39** |
| MB signatures hippocampus |  |  |  | 1.00 | **.53** |
| MB signatures VS |  |  |  |  | 1.00 |

*Notes.* MB signatures, blood oxygenation level dependent signal associated with unique model-based reward prediction error components according to Daw et al. ^4^; vmPFC, ventromedial prefrontal cortex; VS, ventral striatum; *ω*, parameter quantifying the degree of model-based behavior in the two-step task. Correlations with p < .05 in bold

**Table S4.** Mean parameter estimates from the hybrid reinforcement-learning model applied to *n*=93 two-step datasets

| **Parameter** | **Mean (*SD*)** | **95% CI** | |
| --- | --- | --- | --- |
|  |  | **LL** | **UL** |
| $\alpha_{1}$ | 0.42 (0.16) | 0.42 | 0.48 |
| $\alpha_{2}$ | 0.81 (0.19) | 0.77 | 0.85 |
| $\beta_{1}$ | 1.08 (0.50) | 0.98 | 1.18 |
| $\beta_{2}$ | 0.50 (0.14) | 0.47 | 0.53 |
| ω | 0.63 (0.22) | 0.59 | 0.68 |
| $\lambda$ | 0.81 (0.04) | 0.81 | 0.82 |
| $pi$ | 1.05 (0.39) | 0.97 | 1.14 |

*Notes.* Mean, standard deviation (SD) and 95% confidence intervals (CI) of individual parameter estimates (i.e. mean of participants’ posterior parameter distribution resulting from Bayesian model estimation in RStan ^16^) from the hybrid reinforcement-learning model ^4^. Free parameters: $\alpha_{1}$ – first-stage learning rate, $\alpha_{2}$ – second-stage learning rate, $\beta_{1}-$ first-stage inverse temperature, $\beta_{2}$ – second-stage inverse temperature, *ω* –relative balance between model-based and model-free behavior, $\lambda$ - eligibility trace, and $pi$ – perseveration tendency; 95% CI, two-sided 95% confidence interval; LL, lower limit; UL, upper limit

| **Paramete** | ***β*** | ***SE*** | **95% CI** | | ***z*-value** | ***p*** |
| --- | --- | --- | --- | --- | --- | --- |
|  |  |  | **LL** | **UL** |  |  |
| Intercept | 1.17 | 0.07 | 1.06 | 1.27 | 17.81 | **< .001** |
| Correct | 0.96 | 0.07 | 0.85 | 1.07 | 14.74 | **< .001** |
| Outcome | 0.66 | 0.06 | 0.56 | 0.77 | 11.17 | **< .001** |
| Transition | -0.31 | 0.06 | -0.42 | -0.21 | -5.06 | **< .001** |
| Outcome × transition | 1.35 | 0.13 | 1.14 | 1.56 | 10.23 | **< .001** |

**Table S5**. Mean parameter estimates from the mixed-effects logistic regression model applied to *n*=93 two-step datasets

*Notes*. Fixed effects coefficients (*β*) from mixed-effects logistic regression analysis of stay/switch behavior in consecutive trials. *SE*, standard error of the mean; 95% CI, two-sided 95% profile confidence interval; LL, lower limit; UL, upper limit

**
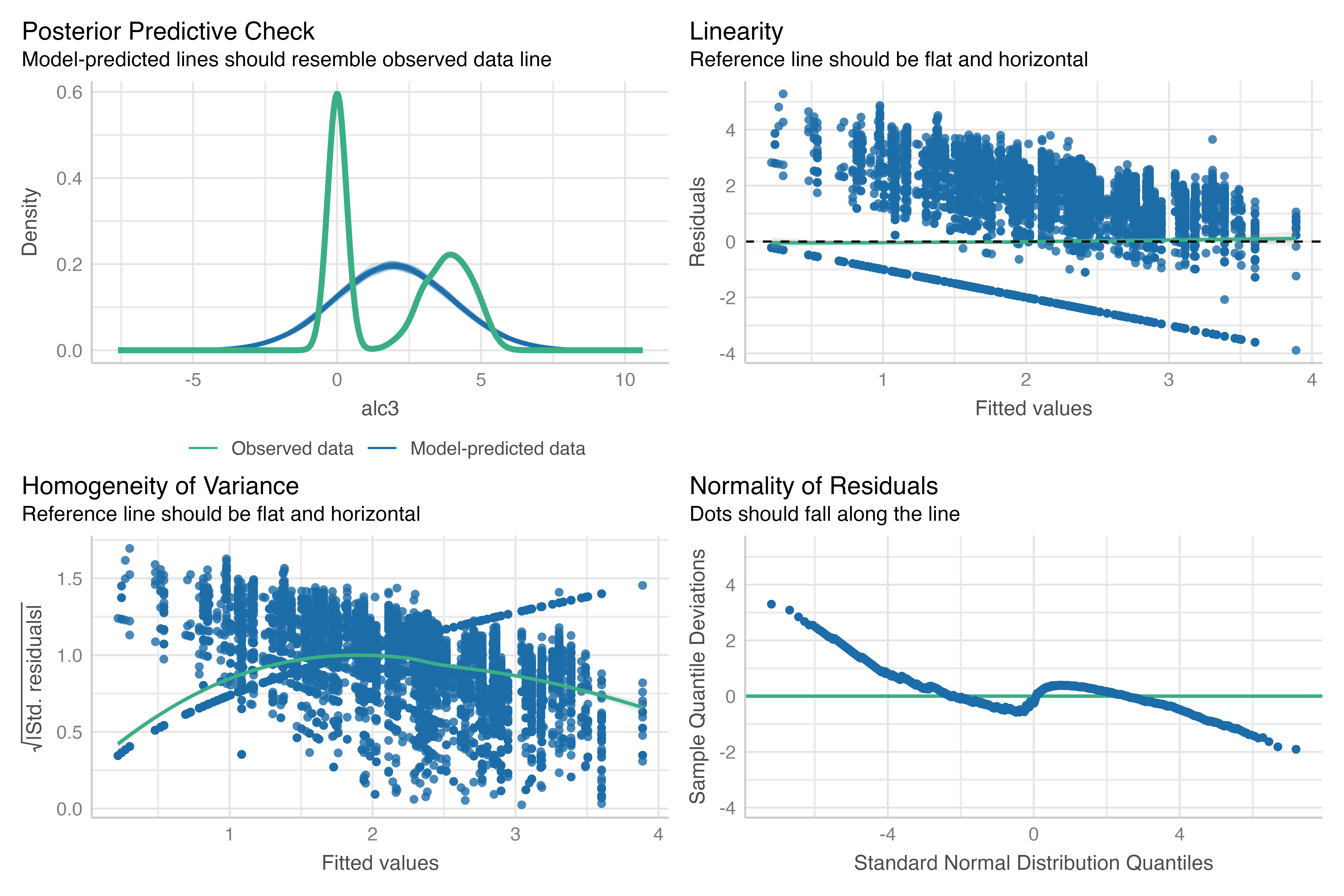
**

**Figure S2.** Model assumptions for linear mixed-effects regression model predicting log daily alcohol consumption in *n*=67 participants with alcohol use disorder by weekly drinking intentions, model-based behavior (*ω*) in the two-step task, and model-based neural signatures.

Data simulation and parameter recovery

To examine parameter reliability of the reinforcement learning model, we performed a parameter recovery analysis based on 1000 simulated datasets. To generate each dataset, we randomly drew a set of seven parameter values from a normal distribution with mean and standard deviation equaling the unconstrained group-level posterior distribution obtained from fitting the model to our empirical dataset. With these known parameter values, we simulated choice data for 201 trials according to the hybrid reinforcement learning model ^4^. Rewards associated with each second-stage action followed the Gaussian random walks implemented in our task. Model estimation based on simulated data was identical to the procedure described above for the empirical data. We report bivariate Pearson-correlations between estimated parameter values and “true”, i.e. known data generating parameters as a metric for parameter recovery.

**Table S6.** Parameter recovery of the hybrid reinforcement learning model based on *n*=1000 simulated datasets

| **Parameter** | $\boldsymbol{\alpha}_{\boldsymbol{1}}$ | $\boldsymbol{\alpha}_{\boldsymbol{2}}$ | $\boldsymbol{\beta}_{\boldsymbol{1}}$ | $\boldsymbol{\beta}_{\boldsymbol{2}}$ | ***ω*** | $\boldsymbol{\lambda}$ | $\boldsymbol{pi}$ |
| --- | --- | --- | --- | --- | --- | --- | --- |
| *r* | 0.55 | 0.73 | 0.88 | 0.88 | 0.82 | 0.05 | 0.74 |

*Notes.* Parameter recovery analysis was based on 1000 simulated datasets and assessed by regressing the estimated parameter values on the ‘true’, i.e. known data-generating parameters. The *r*^2^ metric quantifies parameter recovery. Estimated model parameters: $\alpha_{1}$ - first-stage learning rate, $\alpha_{2}$ – second-stage learning rate, $\beta_{1}-$ first-stage inverse temperature, $\beta_{2}$ – second-stage inverse temperature, *ω* – relative balance between model-based and model-free behavior, $\lambda$ – eligibility trace, and $pi$ – perseveration tendency

**Table S7.** Whole-brain model-based neural signatures in *n* = 86 participants

| **Brain region** | **Cluster size [voxel]** | ***Z*-value** | ***p*_FWE_** | **MNI coordinates** | | |
| --- | --- | --- | --- | --- | --- | --- |
|  |  |  |  | **x** | **y** | **z** |
| Hippocampus L | 321 | > 8.00 | <.001 | -28 | -20 | -16 |
| Hippocampus L |  | 7.16 | <.001 | -30 | -32 | -12 |
| Superior frontal gyrus, medial orbital R | 2601 | > 8.00 | <.001 | 2 | 46 | -10 |
| Anterior cingulate cortex, pregenual L |  | 7.48 | <.001 | -6 | 52 | -2 |
| Olfactory cortex R (incl. caudate nucleus) |  | 7.37 | <.001 | 2 | 22 | -6 |
| Precuneus L | 519 | 7.12 | <.001 | -8 | -52 | 18 |
| Posterior cingulate gyrus L |  | 6.19 | <.001 | -4 | -48 | 30 |
| Middle cingulate & paracingulate gyri L |  | 5.16 | .011 | -8 | -36 | 38 |
| Hippocampus R | 89 | 6.50 | <.001 | 28 | -18 | -16 |
| Hippocampus R |  | 5.32 | .005 | 30 | -10 | -20 |
| Middle temporal gyrus L | 372 | 6.44 | <.001 | -56 | -8 | -16 |
| Middle temporal gyrus L |  | 5.71 | .001 | -56 | 4 | -26 |
| Angular gyrus L | 526 | 6.23 | <.001 | -48 | -70 | 28 |
| Middle occipital gyrus L |  | 6.16 | <.001 | -40 | -78 | 36 |
| Angular gyrus L |  | 5.92 | <.001 | -42 | -62 | 24 |
| Superior temporal gyrus R | 85 | 6.14 | <.001 | 62 | -6 | 2 |
| Rolandic operculum R |  | 5.23 | .008 | 52 | -2 | 10 |
| Middle temporal gyrus R | 174 | 5.84 | <.001 | 60 | -6 | -14 |
| Inferior frontal gyrus pars orbitalis L | 26 | 5.70 | .001 | -34 | 34 | -12 |
| Superior occipital gyrus R | 22 | 5.35 | .004 | 18 | -88 | 32 |
| Middle occipital gyrus R | 16 | 5.33 | .005 | 52 | -72 | 28 |
| Temporal pole: superior temporal gyrus R | 6 | 5.27 | .006 | 54 | 6 | -6 |
| Rolandic operculum R | 9 | 5.25 | .007 | 38 | -2 | 20 |
| Putamen L | 7 | 5.24 | .007 | -12 | 8 | -8 |
| Parahippocampal gyrus R | 9 | 5.23 | .008 | 32 | -30 | -12 |
| Temporal pole: middle temporal gyrus R | 12 | 5.22 | .008 | 44 | 18 | -32 |
| Middle temporal gyrus R | 11 | 5.18 | .010 | 54 | 6 | -30 |
| Middle cingulate & paracingulate gyri L | 10 | 5.15 | .011 | -2 | -16 | 38 |
| Caudate nucleus R | 4 | 5.05 | .018 | 10 | 10 | -8 |
| Olfactory cortex R (incl. putamen) | 5 | 5.04 | .019 | 18 | 8 | -12 |
| Superior frontal gyrus, dorsolateral L | 10 | 4.97 | .025 | -22 | 26 | 42 |
| Rolandic operculum R | 3 | 5.04 | .019 | -48 | -12 | 14 |
| Middle cingulate & paracingulate gyri R | 3 | 5.02 | .020 | 6 | -18 | 40 |
| Insula L | 3 | 4.94 | .028 | -36 | -2 | 16 |
| Postcentral gyrus L | 1 | 4.90 | .035 | -46 | -24 | 66 |

*Notes.* Model-based neural signatures are defined as blood oxygenation level dependent signal associated with unique model-based reward prediction error components according to Daw et al. ^4^. FWE, family-wise error-corrected; L, left; MNI, Montreal Neurological Institute; R, right.

**Table S8.** Whole-brain model-free neural signatures in *n* = 86 participants

| **Brain region** | **Cluster size [voxel]** | ***Z*-value** | ***p*_FWE_** | **MNI coordinates** | | |
| --- | --- | --- | --- | --- | --- | --- |
|  |  |  |  | **x** | **y** | **z** |
| Precentral gyrus L | 10742 | > 8.00 | <.001 | -24 | -8 | 34 |
| Angular gyrus L |  | 7.80 | <.001 | -30 | -48 | 26 |
| Inferior parietal gyrus, excluding Supramarginal and angular gyri L |  | 7.57 | <.001 | -28 | -34 | 32 |
| Caudate nucleus R | 22040 | > 8.00 | <.001 | 24 | 32 | 12 |
| Caudate nucleus R |  | > 8.00 | <.001 | 24 | 2 | 26 |
| Postcentral gyrus R |  | > 8.00 | <.001 | 30 | -36 | 28 |
| Cuneus L | 763 | 7.00 | <.001 | -6 | -94 | 20 |
| Cuneus R |  | 5.60 | 0.001 | 12 | -94 | 18 |
| Superior frontal gyrus, medial L | 53 | 6.20 | <.001 | -10 | 58 | 24 |
| Superior frontal gyrus, dorsolateral L | 178 | 5.85 | <.001 | -14 | 36 | 46 |
| Superior frontal gyrus, dorsolateral L |  | 5.61 | 0.001 | -14 | 34 | 38 |
| Superior frontal gyrus, medial L |  | 5.52 | 0.002 | -10 | 48 | 40 |
| Superior frontal gyrus, dorsolateral R | 15 | 5.82 | <.001 | 18 | 22 | 38 |
| Caudate nucleus R | 15 | 5.79 | <.001 | 10 | 6 | -8 |
| Inferior temporal gyrus R | 48 | 5.74 | 0.001 | 58 | -18 | -32 |
| Angular gyrus L | 52 | 5.37 | 0.004 | -44 | -72 | 30 |
| Precuneus L | 32 | 5.31 | 0.005 | -4 | -62 | 66 |
| Putamen L | 4 | 5.28 | 0.006 | -12 | 6 | -10 |
| Posterior cingulate gyrus L | 7 | 5.21 | 0.008 | -4 | -36 | 16 |
| Superior frontal gyrus, dorsolateral R | 8 | 5.11 | 0.012 | 16 | 36 | 54 |
| Middle cingulate & paracingulate gyri R | 7 | 5.03 | 0.017 | 10 | -2 | 44 |
| Posterior cingulate gyrus L | 7 | 4.98 | 0.022 | -2 | -46 | 28 |
| Middle temporal gyrus L | 10 | 4.98 | 0.022 | -56 | -56 | 18 |
| Lobule IV, V of vermis | 5 | 4.97 | 0.023 | 2 | -48 | 0 |
| Superior frontal gyrus, medial L | 4 | 4.93 | 0.028 | 0 | 50 | 48 |
| Middle frontal gyrus L | 6 | 4.92 | 0.028 | -28 | 26 | 50 |
| Middle frontal gyrus L | 4 | 4.91 | 0.030 | -36 | 18 | 54 |
| Lateral orbital gyrus L | 9 | 4.90 | 0.031 | -38 | 48 | -12 |
| Angular gyrus L | 4 | 4.90 | 0.031 | -52 | -62 | 30 |
| Thalamus, ventral lateral nucelus L | 2 | 5.11 | 0.012 | -16 | -6 | 10 |
| Calcarine fissure and surrounding Cortex L | 2 | 4.97 | 0.023 | 6 | -92 | -16 |
| Posterior cingulate gyrus R | 2 | 4.91 | 0.030 | 6 | -34 | 16 |
| Posterior cingulate gyrus R | 1 | 4.86 | 0.038 | 8 | -36 | 14 |
| Superior frontal gyrus, medial orbital L | 2 | 4.85 | 0.038 | -8 | 42 | -14 |
| Middle cingulate & paracingulate gyri L | 1 | 4.83 | 0.043 | -20 | -30 | 42 |
| Superior frontal gyrus, medial orbital R | 2 | 4.81 | 0.045 | 2 | 48 | -4 |
| Middle temporal gyrus L | 1 | 4.80 | 0.048 | -68 | -28 | -12 |
| Superior frontal gyrus, dorsolateral L | 1 | 4.79 | 0.049 | -16 | 28 | 60 |

*Notes.* Model-free neural signatures, defined as blood oxygenation level dependent signal associated with the model-free reward prediction error component according to Daw et al. ^4^. FWE, family-wise error-corrected; L, left; MNI, Montreal Neurological Institute; R, right.


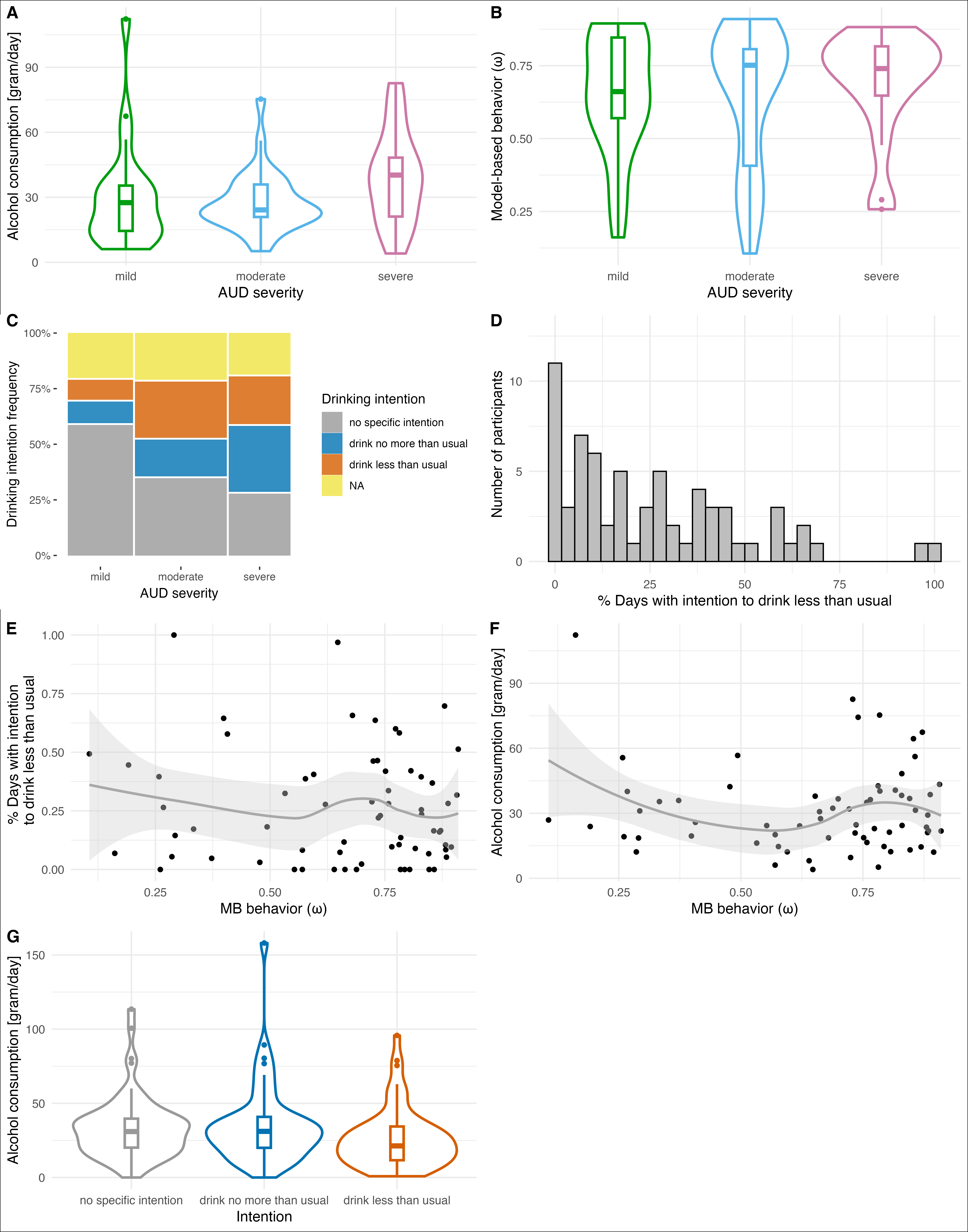


**Figure S3.** Associations between drinking intentions, model-based (MB) behavior ($\boldsymbol{\omega}$), alcohol use disorder (AUD) severity (mild: 2-3 criteria, *n*=21; moderate: 4-5 criteria, *n*=29; severe: 6-7 criteria, *n*=17), and alcohol consumption [gram/day] in the ecological momentary assessment (EMA) sample (*n*=67) with complete EMA data for 11572 days across all participants (except panel C). (A) Linear regression revealed no differences in average daily alcohol intake when comparing individuals with moderate vs. mild (*β*=-2.17, *t*(64) = -0.38, *p* = .704) and severe vs. mild AUD (*β*=8.93, *t*(64) = 1.38, *p* = .171), respectively. This is in line with absolute alcohol consumption not being a diagnostic criterion for AUD according to the Diagnostic and Statistical Manual of Mental Disorders (DSM)-5) ^3^. (B) Linear regression showed no differences in MB behavior when comparing participants with ‘moderate’ vs. ‘mild’ AUD (*β*=-0.01, *t*(64)=-0.08, *p*=.934) or ‘severe‘ vs. ‘mild’ AUD (*β*=0.04, *t*(64)=0.52, *p*=.604), respectively. (C) We found differences in drinking intention frequency by AUD severity (*χ^2^*(4) = 1457, *p* < .001), with the intention to ‘drink less than usual’ being most common in moderate AUD and least common in mild AUD. Based on EMA data for 14579 days with alcohol consumption data across all participants. NA-values in drinking intention thus represent days with data on alcohol consumption, but not on drinking intention. These days were excluded from all other analyses. (D) Distribution of individual percentages of days with the intention to reduce intake out of all days with valid EMA data per participant. (E) We found no correlation between MB behavior ($\boldsymbol{\omega}$) and individual percentages of days with the intention to reduce intake out of all days with valid EMA data ($\boldsymbol{\rho}$=-0.05, *p*=.659). (F) We found no correlation between MB behavior ($\boldsymbol{\omega}$) and individual average daily alcohol intake ($\boldsymbol{\rho}$=0.07, *p*=.561). (G) Linear regression revealed no differences in individual average daily alcohol intake in weeks with the intention to ‘drink less than usual’ vs. ‘no specific intention’ (*β*=-7.25, *t*(164) = -1.74, *p* = .084) and in weeks with the intention to ‘drink no more than usual’ vs. ‘no specific intention’ (*β*=1.82, *t*(164) = 0.42, *p* = .674), respectively. Grey shaded areas in panels (E) and (F) indicate 95% confidence intervals. In panels (A), (B), and (G), horizontal lines within boxplots depict the median, lower and upper hinges mark the 25^th^ and 75^th^ percentiles, whiskers reach out to the smallest and largest value up to maximum 150% of the inter-quartile range, outliers beyond that area are shown as dots, and violin plots depict distribution density.

**Table S9.** Linear mixed-effects regression model predicting log daily alcohol consumption in *n*=67 participants with alcohol use disorder by weekly drinking intentions and model-based behavior (*ω)* in the two-step task

| **Fixed effect** | ***β*** | ***SE*** | **95% CI** | | ***t*-value^1^** | ***p*** |
| --- | --- | --- | --- | --- | --- | --- |
|  |  |  | **LL** | **UL** |  |  |
| Intercept | 2.19 | 0.33 | 1.52 | 2.86 | 6.53 | **<.001** |
| Drink no more than usual | 0.44 | 0.26 | -0.09 | 0.98 | 1.65 | .098 |
| Drink less than usual | 0.15 | 0.23 | -0.30 | 0.60 | 0.66 | .510 |
| *ω* | -0.11 | 0.48 | -1.07 | 0.84 | -0.23 | .820 |
| Drink no more than usual × *ω* | -0.60 | 0.38 | -1.37 | 0.16 | -1.60 | .111 |
| Drink less than usual × *ω* | -1.06 | 0.32 | -1.71 | -0.42 | -3.31 | **.001** |

*Notes*. ‘Drink less than usual’ and ‘drink no more than usual’ refer to dummy-coded drinking intention, comparing log daily alcohol consumption on days with this intention to consumption on days with ‘no specific intention’ (reference level), respectively. LL, lower limit; *SE*, standard error of the mean; UL, upper limit; *ω*, parameter quantifying the degree of model-based behavior in the two-step task; 95% CI: two-sided 95% profile confidence interval

^1^ *df* = 11562

**Table S10.** Linear mixed-effects regression model predicting log daily alcohol consumption in *n*=67 participants with alcohol use disorder by weekly drinking intentions and model-based behavior (MB score) in the two-step task

| **Fixed effect** | ***β*** | ***SE*** | **95% CI** | | ***t*-value^1^** | ***p*** |
| --- | --- | --- | --- | --- | --- | --- |
|  |  |  | **LL** | **UL** |  |  |
| Intercept | 2.10 | 0.23 | 1.65 | 2.55 | 9.33 | **<.001** |
| Drink no more than usual | 0.19 | 0.19 | -0.17 | 0.58 | 1.04 | .299 |
| Drink less than usual | -0.13 | 0.16 | -0.44 | 0.19 | -0.84 | .402 |
| MB score | 0.01 | 0.14 | -0.26 | 0.28 | 0.08 | .938 |
| Drink no more than usual × MB score | -0.10 | 0.11 | -0.34 | 0.12 | -0.92 | .358 |
| Drink less than usual × MB score | -0.29 | 0.09 | -0.48 | -0.10 | -3.07 | **.002** |

*Notes*. The MB score was derived from logistic mixed-effects regression. ‘Drink less than usual’ and ‘drink no more than usual’ refer to dummy-coded drinking intention, comparing log daily alcohol consumption on days with this intention to consumption on days with ‘no specific intention’ (reference level), respectively. LL, lower limit; MB, model-based; *SE*, standard error of the mean; UP, upper limit; 95% CI, two-sided 95% profile confidence interval

^1^ *df* = 11562

**Table S11.** Linear mixed-effects regression model predicting log daily alcohol consumption in *n*=67 participants with alcohol use disorder by weekly drinking intentions, model-based behavior ($\omega$) in the two-step task, the proportion of correct matrices from the Raven Standard Progressive Matrices ^10^, the number of correct digits from the Digit Symbol Substitution Test (DSST) ^9^, and the length of the maximum backwards remembered digit span from the Digit Span Backwards Test (DSBW) ^11^.

| **Fixed effect** | ***β*** | ***SE*** | **95% CI** | | ***t*-value^1^** | ***p*** |
| --- | --- | --- | --- | --- | --- | --- |
|  |  |  | **LL** | **UL** |  |  |
| Intercept | 1.94 | 0.35 | 1.25 | 2.63 | 5.56 | **<.001** |
| Drink no more than usual | 0.45 | 0.28 | -0.11 | 1.04 | 1.60 | .110 |
| Drink less than usual | 0.12 | 0.24 | -0.37 | 0.61 | 0.48 | .633 |
| $\omega$ | 0.23 | 0.50 | -0.77 | 1.23 | 0.47 | .642 |
| Raven | -0.16 | 0.11 | -0.37 | 0.05 | -1.51 | .131 |
| DSST | -0.16 | 0.11 | -0.37 | 0.06 | -1.47 | .141 |
| DSBW | 0.004 | 0.10 | -0.20 | 0.21 | 0.04 | .967 |
| Drink no more than usual × $\omega$ | -0.65 | 0.41 | -1.49 | 0.17 | -1.58 | .113 |
| Drink less than usual × $\omega$ | -1.02 | 0.35 | -1.72 | -0.31 | -2.90 | **.004** |
| Drink no more than usual × Raven | -0.07 | 0.10 | -0.27 | 0.13 | -0.76 | .447 |
| Drink less than usual × Raven | -0.02 | 0.08 | -0.19 | 0.14 | -0.23 | .815 |
| Drink no more than usual × DSST | -0.04 | 0.10 | -0.25 | 0.15 | -0.45 | .651 |
| Drink less than usual × DSST | 0.05 | 0.08 | -0.12 | 0.22 | 0.58 | .564 |
| Drink no more than usual × DSBW | -0.07 | 0.08 | -0.24 | 0.10 | -0.83 | .404 |
| Drink less than usual × DSBW | 0.05 | 0.07 | -0.10 | 0.20 | 0.67 | .500 |

*Notes*. ‘Drink less than usual’ and ‘drink no more than usual’ refer to dummy-coded drinking intention, comparing log daily alcohol consumption on days with this intention to consumption on days with ‘no specific intention’ (reference level), respectively. DSST, Raven Matrices, and DSBW scores were *z*-standardized. LL, lower limit; *SE*, standard error of the mean; UP, upper limit; *ω*, parameter quantifying the degree of model-based behavior in the two-step task; 95% CI, two-sided 95% profile confidence interval

^1^ *df* = 10476

**Table S12.** Linear mixed-effects regression model predicting log daily alcohol consumption in *n*=67 participants with alcohol use disorder by weekly drinking intentions, model-based behavior (MB score) in the two-step task, and model-based neural signatures

| **Fixed effect** | ***β*** | ***SE*** | **95% CI** | | ***t*-value^1^** | ***p*** |
| --- | --- | --- | --- | --- | --- | --- |
|  |  |  | **LL** | **UL** |  |  |
| Intercept | 2.26 | 0.25 | 1.77 | 2.76 | 9.10 | **<.001** |
| Drink no more than usual | 0.12 | 0.20 | -0.29 | 0.54 | 0.56 | .574 |
| Drink less than usual | -0.14 | 0.17 | -0.48 | 0.21 | -0.78 | .433 |
| MB score | -0.003 | 0.14 | -0.27 | 0.27 | -0.02 | .985 |
| MB signatures hippocampus | -0.16 | 0.52 | -1.19 | 0.86 | -0.31 | .753 |
| MB signatures VS | -0.23 | 0.31 | -0.85 | 0.38 | -0.75 | .452 |
| MB signatures vmPFC | -0.11 | 0.34 | -0.78 | 0.57 | -0.31 | .756 |
| Drink no more than usual × MB score | -0.09 | 0.11 | -0.32 | 0.13 | -0.81 | .416 |
| Drink less than usual × MB score | -0.28 | 0.09 | -0.46 | -0.10 | -3.13 | **.002** |
| Drink no more than usual × MB signatures hippocampus | -0.34 | 0.39 | -1.14 | 0.44 | -0.87 | .383 |
| Drink less than usual × MB signatures hippocampus | -0.69 | 0.33 | -1.35 | -0.04 | -2.12 | **.034** |
| Drink no more than usual × MB signatures VS | 0.57 | 0.25 | 0.18 | 1.08 | 2.29 | **.022** |
| Drink less than usual × MB signatures VS | 0.42 | 0.20 | 0.04 | 0.82 | 2.17 | **.030** |
| Drink no more than usual × MB signatures vmPFC | -0.04 | 0.26 | -0.58 | 0.48 | -0.16 | .871 |
| Drink less than usual × MB signatures vmPFC | 0.06 | 0.22 | -0.38 | 0.51 | 0.28 | .781 |

*Notes*. The MB score was derived from logistic mixed-effects regression. ‘Drink less than usual’ and ‘drink no more than usual’ refer to dummy-coded drinking intention, comparing log daily alcohol consumption on days with this intention to consumption on days with ‘no specific intention’ (reference level), respectively. LL, lower limit; MB signatures, blood oxygenation level dependent signal associated with unique model-based reward prediction error components according to Daw et al. ^4^; *SE*, standard error of the mean; UP, upper limit; vmPFC, ventromedial prefrontal cortex; VS, ventral striatum; 95% CI, two-sided 95% profile confidence interval

^1^ *df* = 11553

**Table S13.** Linear mixed-effects regression model predicting log daily alcohol consumption in *n*=67 participants with alcohol use disorder by weekly drinking intentions, model-based behavior ($\omega$) in the two-step task, model-based neural signatures, the proportion of correct matrices from the Raven Standard Progressive Matrices ^10^, the number of correct digits from the Digit Symbol Substitution Test (DSST) ^9^, and the length of the maximum backwards remembered digit span from the Digit Span Backwards Test (DSBW) ^11^

| **Fixed effect** | ***β*** | ***SE*** | **95% CI** | | ***t*-value^1^** | ***p*** |
| --- | --- | --- | --- | --- | --- | --- |
|  |  |  | **LL** | **UL** |  |  |
| Intercept | 2.10 | 0.35 | 1.41 | 2.80 | 6.01 | **<.001** |
| Drink no more than usual | 0.34 | 0.30 | -0.25 | 0.94 | 1.14 | .252 |
| Drink less than usual | 0.12 | 0.24 | -0.36 | 0.59 | 0.51 | .608 |
| $\omega$ | 0.32 | 0.50 | -0.67 | 1.31 | 0.65 | .516 |
| Raven | -0.20 | 0.11 | -0.41 | 0.01 | -1.88 | .060 |
| DSST | -0.15 | 0.11 | -0.37 | 0.07 | -1.33 | .183 |
| DSBW | -0.05 | 0.10 | -0.26 | 0.16 | -0.52 | .603 |
| MB signatures hippocampus | 0.03 | 0.50 | -0.96 | 1.01 | 0.05 | .958 |
| MB signatures VS | -0.29 | 0.29 | -0.87 | 0.30 | -0.97 | .330 |
| MB signatures vmPFC | -0.37 | 0.33 | -1.03 | 0.30 | -1.10 | .271 |
| Drink no more than usual × $\omega$ | -0.50 | 0.41 | -1.33 | 0.33 | -1.22 | .222 |
| Drink less than usual × $\omega$ | -1.11 | 0.33 | -1.77 | -0.43 | -3.33 | **.001** |
| Drink no more than usual × Raven | -0.11 | 0.10 | -0.32 | 0.10 | -1.06 | .287 |
| Drink less than usual × Raven | -0.03 | 0.08 | -0.20 | 0.13 | -0.37 | .713 |
| Drink no more than usual × DSST | -0.06 | 0.10 | -0.27 | 0.14 | -0.62 | .534 |
| Drink less than usual × DSST | 0.06 | 0.08 | -0.10 | 0.23 | 0.71 | .478 |
| Drink no more than usual × DSBW | -0.05 | 0.09 | -0.23 | 0.12 | -0.59 | .553 |
| Drink less than usual × DSBW | 0.11 | 0.07 | -0.04 | 0.26 | 1.43 | .154 |
| Drink no more than usual × MB signatures hippocampus | -0.31 | 0.40 | -1.13 | 0.50 | -0.77 | .440 |
| Drink less than usual × MB signatures hippocampus | -0.86 | 0.33 | -1.52 | -0.19 | -2.57 | **.010** |
| Drink no more than usual × MB signatures VS | 0.55 | 0.25 | 0.06 | 1.07 | 2.22 | **.027** |
| Drink less than usual × MB signatures VS | 0.45 | 0.20 | 0.06 | 0.85 | 2.31 | **.021** |
| Drink no more than usual × MB signatures vmPFC | -0.22 | 0.29 | -0.82 | 0.37 | -0.73 | .462 |
| Drink less than usual × MB signatures vmPFC | 0.28 | 0.24 | -0.21 | 0.76 | 0.14 | .253 |

*Notes*. ‘Drink less than usual’ and ‘drink no more than usual’ refer to dummy-coded drinking intention, comparing log daily alcohol consumption on days with this intention to consumption on days with ‘no specific intention’ (reference level), respectively. DSST, Raven Matrices, and DSBW scores were *z*-standardized. LL, lower limit; MB signatures, blood oxygenation level dependent signal associated with unique model-based reward prediction error components according to Daw et al. ^4^; *SE*, standard error of the mean; UP, upper limit; vmPFC, ventromedial prefrontal cortex; VS, ventral striatum; *ω*, parameter quantifying the degree of model-based behavior in the two-step task; 95% CI, two-sided 95% profile confidence interval

^1^ *df* = 10467

**Table S14.** Robust linear mixed-effects regression model predicting log daily alcohol consumption in *n*=67 participants with alcohol use disorder by weekly drinking intentions and model-based behavior (*ω)* in the two-step task

| **Fixed effect** | ***β*** | ***SE*** | ***t*-value^1^** | ***p*** |
| --- | --- | --- | --- | --- |
| Intercept | 2.17 | 0.40 | 5.44 | **<.001** |
| Drink no more than usual | 0.47 | 0.25 | 1.93 | .054 |
| Drink less than usual | 0.20 | 0.23 | 0.85 | .395 |
| *ω* | -0.13 | 0.57 | -0.22 | .825 |
| Drink no more than usual × *ω* | -0.68 | 0.35 | -1.94 | .053 |
| Drink less than usual × *ω* | -1.20 | 0.33 | -3.66 | **<.001** |

*Notes.* ‘Drink less than usual’ and ‘drink no more than usual’ refer to dummy-coded drinking intention, comparing log daily alcohol consumption on days with this intention to consumption on days with ‘no specific intention’ (reference level), respectively. LL, lower limit; *SE*, standard error of the mean; UP, upper limit; *ω*, parameter quantifying the degree of model-based behavior in the two-step task; 95% CI: two-sided 95% profile confidence interval

^1^ *df* = 11562

**Table S15.** Robust linear mixed-effects regression model predicting log daily alcohol consumption in *n*=67 participants with alcohol use disorder by weekly drinking intentions and model-based behavior (MB score) in the two-step task

| **Fixed effect** | ***β*** | ***SE*** | ***t*-value^1^** | ***p*** |
| --- | --- | --- | --- | --- |
| Intercept | 2.07 | 0.27 | 7.73 | **<.001** |
| Drink no more than usual | 0.21 | 0.18 | 1.16 | .247 |
| Drink less than usual | -0.14 | 0.16 | -0.89 | .375 |
| MB score | 0.01 | 0.16 | 0.07 | .948 |
| Drink no more than usual × MB score | -0.12 | 0.11 | -1.07 | .283 |
| Drink less than usual × MB score | -0.31 | 0.10 | -3.21 | **.001** |

*Notes*. The MB score was derived from logistic mixed-effects regression. ‘Drink less than usual’ and ‘drink no more than usual’ refer to dummy-coded drinking intention, comparing log daily alcohol consumption on days with this intention to consumption on days with ‘no specific intention’ (reference level), respectively. LL, lower limit; MB, model-based; *SE*, standard error of the mean; UP, upper limit; 95% CI, two-sided 95% profile confidence interval

^1^ *df* = 11562

**Table S16.** Robust linear mixed-effects regression model predicting log daily alcohol consumption in *n*=67 participants with alcohol use disorder by weekly drinking intentions, model-based behavior ($\omega$) in the two-step task, the proportion of correct matrices from the Raven Standard Progressive Matrices ^10^, the number of correct digits from the Digit Symbol Substitution Test (DSST) ^9^, and the length of the maximum backwards remembered digit span from the Digit Span Backwards Test (DSBW) ^11^

| **Fixed effect** | ***β*** | ***SE*** | ***t*-value^1^** | ***p*** |
| --- | --- | --- | --- | --- |
|  |  |  |  |  |
| Intercept | 1.93 | 0.43 | 4.47 | **<.001** |
| Drink no more than usual | 0.36 | 0.23 | 1.53 | .125 |
| Drink less than usual | 0.16 | 0.23 | 0.69 | .489 |
| $\omega$ | 0.23 | 0.63 | 0.36 | .716 |
| Raven | -0.17 | 0.13 | -1.29 | .197 |
| DSST | -0.18 | 0.13 | -1.33 | .183 |
| DSBW | 0.01 | 0.13 | 0.08 | .938 |
| Drink no more than usual × $\omega$ | -0.51 | 0.33 | -1.52 | .127 |
| Drink less than usual × $\omega$ | -1.15 | 0.34 | -3.42 | **<.001** |
| Drink no more than usual × Raven | -0.15 | 0.08 | -1.85 | .065 |
| Drink less than usual × Raven | 0.01 | 0.08 | 0.07 | .943 |
| Drink no more than usual × DSST | 0.02 | 0.08 | 0.27 | .786 |
| Drink less than usual × DSST | 0.03 | 0.08 | 0.36 | .719 |
| Drink no more than usual × DSBW | -0.06 | 0.07 | -0.92 | .356 |
| Drink less than usual × DSBW | 0.08 | 0.07 | 1.20 | .231 |

*Notes*. ‘Drink less than usual’ and ‘drink no more than usual’ refer to dummy-coded drinking intention, comparing log daily alcohol consumption on days with this intention to consumption on days with ‘no specific intention’ (reference level), respectively. DSST, Raven Matrices, and DSBW scores were *z*-standardized. LL, lower limit; *SE*, standard error of the mean; UP, upper limit; *ω*, parameter quantifying the degree of model-based behavior in the two-step task; 95% CI, two-sided 95% profile confidence interval

^1^ *df* = 10476

**Table S17.** Robust linear mixed-effects regression model predicting log daily alcohol consumption in n=67 participants with alcohol use disorder by weekly drinking intentions, model-based behavior (ω) in the two-step task, and model-based neural signatures

| **Fixed effect** | ***β*** | ***SE*** | ***t*-value^1^** | ***p*** |
| --- | --- | --- | --- | --- |
| Intercept | 2.38 | 0.43 | 5.59 | **<.001** |
| Drink no more than usual | 0.32 | 0.28 | 1.13 | .257 |
| Drink less than usual | 0.20 | 0.24 | 0.81 | .417 |
| *ω* | -0.20 | 0.59 | -0.34 | .735 |
| MB signatures hippocampus | -0.19 | 0.63 | -0.31 | .760 |
| MB signatures VS | -0.25 | 0.38 | -0.67 | .501 |
| MB signatures vmPFC | -0.12 | 0.42 | -0.30 | .766 |
| Drink no more than usual × *ω* | -0.57 | 0.38 | -1.51 | .131 |
| Drink less than usual × *ω* | -1.22 | 0.32 | -3.76 | **<.001** |
| Drink no more than usual × MB signatures hippocampus | -0.32 | 0.38 | -0.84 | .401 |
| Drink less than usual × MB signatures hippocampus | -0.85 | 0.34 | -2.52 | **.012** |
| Drink no more than usual × MB signatures VS | 0.60 | 0.24 | 2.47 | **.014** |
| Drink less than usual × MB signatures VS | 0.45 | 0.20 | 2.26 | **.024** |
| Drink no more than usual × MB signatures vmPFC | 0.001 | 0.26 | 0.003 | .998 |
| Drink less than usual × MB signatures vmPFC | 0.16 | 0.23 | 0.69 | .490 |

*Notes.* ‘Drink less than usual’ and ‘drink no more than usual’ refer to dummy-coded drinking intention, comparing log daily alcohol consumption on days with this intention to consumption on days with ‘no specific intention’ (reference level), respectively. MB signatures, blood oxygenation level dependent signal associated with unique model-based reward prediction error components according to Daw et al. ^4^; SE, standard error of the mean; vmPFC, ventromedial prefrontal cortex; VS, ventral striatum; ω, parameter quantifying the degree of model-based behavior in the two-step task

^1^ *df* = 11553

**Table S18.** Robust linear mixed-effects regression model predicting log daily alcohol consumption in *n*=67 participants with alcohol use disorder by weekly drinking intentions, model-based behavior (MB score) in the two-step task, and model-based neural signatures

| **Fixed effect** | $\boldsymbol{\beta}$ | ***SE*** | ***t*-value^1^** | ***p*** |
| --- | --- | --- | --- | --- |
| Intercept | 2.26 | 0.30 | 7.50 | **<.001** |
| Drink no more than usual | 0.12 | 0.21 | 0.55 | .581 |
| Drink less than usual | -0.13 | 0.19 | -0.70 | .487 |
| MB score | -0.003 | 0.17 | -0.02 | .985 |
| MB signatures hippocampus | -0.19 | 0.63 | -0.30 | .762 |
| MB signatures VS | -0.24 | 0.38 | -0.64 | .519 |
| MB signatures vmPFC | -0.14 | 0.42 | -0.33 | .742 |
| Drink no more than usual × MB score | -0.11 | 0.11 | -0.98 | .325 |
| Drink less than usual × MB score | -0.32 | 0.10 | -3.23 | **.001** |
| Drink no more than usual × MB signatures hippocampus | -0.30 | 0.40 | -0.76 | .449 |
| Drink less than usual × MB signatures hippocampus | -0.77 | 0.36 | -2.16 | **.031** |
| Drink no more than usual × MB signatures VS | 0.64 | 0.25 | 2.54 | **.011** |
| Drink less than usual × MB signatures VS | 0.51 | 0.21 | 2.41 | **.016** |
| Drink no more than usual × MB signatures vmPFC | -0.05 | 0.27 | -0.20 | .841 |
| Drink less than usual × MB signatures vmPFC | 0.04 | 0.24 | 0.17 | .866 |

*Notes*: The MB score was derived from logistic mixed-effects regression. ‘Drink less than usual’ and ‘drink no more than usual’ refer to dummy-coded drinking intention, comparing log daily alcohol consumption on days with this intention to consumption on days with ‘no specific intention’ (reference level), respectively. MB signatures, blood oxygenation level dependent signal associated with unique model-based reward prediction error components according to Daw et al. ^4^; *SE*, standard error of the mean; vmPFC, ventromedial prefrontal cortex; VS, ventral striatum

^1^ *df* = 11553

**Table S19.** Robust linear mixed-effects regression model predicting log daily alcohol consumption in *n*=67 participants with alcohol use disorder by weekly drinking intentions, model-based behavior ($\omega$) in the two-step task, model-based neural signatures, the proportion of correct matrices from the Raven Standard Progressive Matrices ^10^, the number of correct digits from the Digit Symbol Substitution Test (DSST) ^9^, and the length of the maximum backwards remembered digit span from the Digit Span Backwards Test (DSBW) ^11^

| **Fixed effect** | ***β*** | ***SE*** | ***t*-value^1^** | ***p*** |
| --- | --- | --- | --- | --- |
|  |  |  |  |  |
| Intercept | 2.10 | 0.45 | 4.67 | **<.001** |
| Drink no more than usual | 0.26 | 0.25 | 1.07 | .285 |
| Drink less than usual | 0.17 | 0.21 | 0.83 | .409 |
| $\omega$ | 0.37 | 0.64 | 0.57 | .568 |
| Raven | -0.21 | 0.14 | -1.57 | .116 |
| DSST | -0.18 | 0.14 | -1.25 | .212 |
| DSBW | -0.05 | 0.13 | -0.40 | .688 |
| MB signatures hippocampus | 0.01 | 0.65 | 0.02 | .987 |
| MB signatures VS | -0.31 | 0.38 | -0.82 | .411 |
| MB signatures vmPFC | -0.41 | 0.43 | -0.94 | .345 |
| Drink no more than usual × $\omega$ | -0.42 | 0.34 | -1.25 | .210 |
| Drink less than usual × $\omega$ | -1.28 | 0.30 | -4.26 | **<.001** |
| Drink no more than usual × Raven | -0.17 | 0.09 | -1.94 | .053 |
| Drink less than usual × Raven | -0.0003 | 0.08 | -0.003 | .997 |
| Drink no more than usual × DSST | -0.0004 | 0.08 | -0.01 | .996 |
| Drink less than usual × DSST | 0.03 | 0.08 | 0.41 | .679 |
| Drink no more than usual × DSBW | -0.05 | 0.07 | -0.69 | .489 |
| Drink less than usual × DSBW | 0.15 | 0.07 | 2.27 | **.023** |
| Drink no more than usual × MB signatures hippocampus | -0.33 | 0.34 | -0.98 | .326 |
| Drink less than usual × MB signatures hippocampus | -0.96 | 0.31 | -3.08 | **.002** |
| Drink no more than usual × MB signatures VS | 0.48 | 0.22 | 2.23 | **.025** |
| Drink less than usual × MB signatures VS | 0.53 | 0.19 | 2.83 | **.005** |
| Drink no more than usual × MB signatures vmPFC | -0.13 | 0.25 | -0.50 | .617 |
| Drink less than usual × MB signatures vmPFC | 0.33 | 0.23 | 1.45 | .148 |

*Notes*: ‘Drink less than usual’ and ‘drink no more than usual’ refer to dummy-coded drinking intention, comparing log daily alcohol consumption on days with this intention to consumption on days with ‘no specific intention’ (reference level), respectively. DSST, Raven Matrices, and DSBW scores were *z*-standardized. MB signatures, blood oxygenation level dependent signal associated with unique model-based reward prediction error components according to Daw et al. ^4^; *SE*, standard error of the mean; vmPFC, ventromedial prefrontal cortex; VS, ventral striatum, *ω*, parameter quantifying the degree of model-based behavior in the two-step task
^1^ *df* = 10467

**Table S20.** Logistic mixed-effects regression model predicting the probability of drinking (vs. abstinence) on a given day in *n*=67 participants with alcohol use disorder by weekly drinking intentions and model-based behavior ($\omega$) in the two-step task

| **Fixed effect** | **Odds Ratios** | ***SE*** | **95% CI** | | **z-value** | ***p*** |
| --- | --- | --- | --- | --- | --- | --- |
|  |  |  | **LL** | **UL** |  |  |
| Intercept | 1.55 | 0.62 | -0.35 | 1.22 | 1.09 | .275 |
| Drink no more than usual | 1.76 | 0.60 | -0.10 | 1.23 | 1.68 | .093 |
| Drink less than usual | 1.16 | 0.35 | -0.44 | 0.73 | 0.50 | .620 |
| *ω* | 0.73 | 0.42 | -1.44 | 0.80 | -0.56 | .576 |
| Drink no more than usual × *ω* | 0.48 | 0.23 | -1.68 | 0.21 | -1.52 | .128 |
| Drink less than usual × *ω* | 0.31 | 0.13 | -2.01 | -0.35 | -2.78 | **.005** |

*Notes*. ‘Drink less than usual’ and ‘drink no more than usual’ refer to dummy-coded drinking intention, comparing the probability of drinking on days with this intention to the probability of drinking on days with ‘no specific intention’ (reference level), respectively. LL, lower limit; *SE*, standard error of the mean; UP, upper limit; *ω*, parameter quantifying the degree of model-based behavior in the two-step task; 95% CI, two-sided 95% profile confidence interval

**Table S21.** Logistic mixed-effects regression model predicting the probability of drinking (vs. abstinence) on a given day in *n*=67 participants with alcohol use disorder by weekly drinking intentions, model-based behavior ($\omega$) in the two-step task, the proportion of correct matrices from the Raven Standard Progressive Matrices ^10^, the number of correct digits from the Digit Symbol Substitution Test (DSST) ^9^, and the length of the maximum backwards remembered digit span from the Digit Span Backwards Test (DSBW) ^11^

| **Fixed effect** | ***Odds Ratios*** | ***SE*** | **95% CI** | | **z-value** | ***p*** |
| --- | --- | --- | --- | --- | --- | --- |
|  |  |  | **LL** | **UL** |  |  |
| Intercept | 1.21 | 0.52 | -0.65 | 1.03 | 0.44 | .657 |
| Drink no more than usual | 1.70 | 0.61 | -0.17 | 1.23 | 1.48 | .140 |
| Drink less than usual | 1.01 | 0.33 | -0.62 | 0.65 | 0.04 | .968 |
| $\omega$ | 1.04 | 0.64 | -1.17 | 1.25 | 0.06 | .950 |
| Raven | 0.83 | 0.11 | -0.44 | 0.06 | -1.49 | .137 |
| DSST | 0.87 | 0.12 | -0.40 | 0.12 | -1.02 | .306 |
| DSBW | 1.00 | 0.13 | -0.26 | 0.25 | -0.03 | .977 |
| Drink no more than usual × $\omega$ | 0.48 | 0.25 | -1.75 | 0.28 | -1.42 | .156 |
| Drink less than usual × $\omega$ | 0.37 | 0.17 | -1.90 | -0.08 | -2.13 | **.033** |
| Drink no more than usual × Raven | 0.92 | 0.11 | -0.32 | 0.16 | -0.66 | .507 |
| Drink less than usual × Raven | 0.91 | 0.10 | -0.31 | 0.11 | -0.90 | .370 |
| Drink no more than usual × DSST | 0.93 | 0.12 | -0.32 | 0.17 | -0.59 | .558 |
| Drink less than usual × DSST | 1.07 | 0.12 | -0.14 | 0.29 | 0.64 | .520 |
| Drink no more than usual × DSBW | 0.89 | 0.09 | -0.32 | 0.09 | -1.09 | .274 |
| Drink less than usual × DSBW | 0.99 | 0.10 | -0.20 | 0.19 | -0.07 | .941 |

*Notes*. ‘Drink less than usual’ and ‘drink no more than usual’ refer to dummy-coded drinking intention, comparing the probability of drinking on days with this intention to the probability of drinking on days with ‘no specific intention’ (reference level), respectively. DSST, Raven Matrices, and DSBW scores were *z*-standardized. LL, lower limit; *SE*, standard error of the mean; UP, upper limit; *ω*, parameter quantifying the degree of model-based behavior in the two-step task; 95% CI, two-sided 95% Wald confidence interval

**Table S22.** Logistic mixed-effects regression model predicting the probability of drinking (vs. abstinence) on a given day in *n*=67 participants with alcohol use disorder by weekly drinking intentions, model-based behavior ($\omega$) in the two-step task, model-based neural signatures, the proportion of correct matrices from the Raven Standard Progressive Matrices ^10^, the number of correct digits from the Digit Symbol Substitution Test (DSST) ^9^, and the length of the maximum backwards remembered digit span from the Digit Span Backwards Test (DSBW) ^11^

| **Fixed effect** | ***Odds Ratios*** | ***SE*** | **95% CI** | | ***z-*value** | ***p*** |
| --- | --- | --- | --- | --- | --- | --- |
|  |  |  | **LL** | **UL** |  |  |
| Intercept | 1.47 | 0.62 | -0.45 | 1.22 | 0.91 | .364 |
| Drink no more than usual | 1.60 | 0.59 | -0.25 | 1.19 | 1.28 | .199 |
| Drink less than usual | 1.01 | 0.32 | -0.62 | 0.63 | 0.02 | .986 |
| $\omega$ | 1.19 | 0.72 | -1.01 | 1.35 | 0.28 | .777 |
| Raven | 0.79 | 0.10 | -0.49 | 0.01 | -1.86 | .063 |
| DSST | 0.88 | 0.12 | -0.39 | 0.14 | -0.93 | .352 |
| DSBW | 0.93 | 0.12 | -0.33 | 0.17 | -0.60 | .545 |
| MB signatures hippocampus | 1.15 | 0.69 | -1.04 | 1.31 | 0.23 | .817 |
| MB signatures VS | 0.68 | 0.24 | -1.08 | 0.31 | -1.08 | .280 |
| MB signatures vmPFC | 0.59 | 0.24 | -1.32 | 0.28 | -1.28 | .200 |
| Drink no more than usual × $\omega$ | 0.60 | 0.30 | -1.51 | 0.48 | -1.01 | .310 |
| Drink less than usual × $\omega$ | 0.34 | 0.16 | -1.95 | -0.18 | -2.36 | **.018** |
| Drink no more than usual × Raven | 0.84 | 0.11 | -0.43 | 0.08 | -1.36 | .175 |
| Drink less than usual × Raven | 0.90 | 0.10 | -0.33 | 0.11 | -0.99 | .324 |
| Drink no more than usual × DSST | 0.88 | 0.11 | -0.37 | 0.12 | -0.99 | .324 |
| Drink less than usual × DSST | 1.08 | 0.12 | -0.14 | 0.29 | 0.69 | .489 |
| Drink no more than usual × DSBW | 0.87 | 0.10 | -0.35 | 0.08 | -1.24 | .213 |
| Drink less than usual × DSBW | 1.06 | 0.11 | -0.14 | 0.26 | 0.58 | .564 |
| Drink no more than usual × MB signatures hippocampus | 0.73 | 0.36 | -1.28 | 0.65 | -0.65 | .519 |
| Drink less than usual × MB signatures hippocampus | 0.40 | 0.18 | -1.78 | -0.04 | -2.06 | **.039** |
| Drink no more than usual × MB signatures VS | 1.89 | 0.58 | 0.04 | 1.24 | 2.09 | **.037** |
| Drink less than usual × MB signatures VS | 1.74 | 0.45 | 0.05 | 1.06 | 2.15 | **.031** |
| Drink no more than usual × MB signatures vmPFC | 0.57 | 0.21 | -1.28 | 0.16 | -1.51 | .130 |
| Drink less than usual × MB signatures vmPFC | 1.30 | 0.43 | -0.38 | 0.90 | 0.79 | .428 |

*Notes*: ‘Drink less than usual’ and ‘drink no more than usual’ refer to dummy-coded drinking intention, comparing log daily alcohol consumption on days with this intention to consumption on days with ‘no specific intention’ (reference level), respectively. DSST, Raven Matrices, and DSBW scores were *z*-standardized. MB signatures, blood oxygenation level dependent signal associated with unique model-based reward prediction error components according to Daw et al. ^4^; *SE*, standard error of the mean; vmPFC, ventromedial prefrontal cortex; VS, ventral striatum, *ω*, parameter quantifying the degree of model-based behavior in the two-step task; 95% CI, two-sided 95% Wald confidence interval

**Table S23.** Logistic mixed-effects regression model predicting the probability of drinking (vs. abstinence) on a given day in *n*=67 participants with alcohol use disorder by weekly drinking intentions and model-based behavior (MB score) in the two-step task

| **Fixed effect** | ***Odds Ratios*** | ***SE*** | **95% CI** | | **z-value** | ***p*** |
| --- | --- | --- | --- | --- | --- | --- |
|  |  |  | **LL** | **UL** |  |  |
| Intercept | 1.33 | 0.36 | -0.24 | 0.82 | 1.06 | .290 |
| Drink no more than usual | 1.31 | 0.31 | -0.19 | 0.73 | 1.16 | .246 |
| Drink less than usual | 0.88 | 0.18 | -0.54 | 0.27 | -0.64 | .521 |
| MB score | 0.96 | 0.16 | -0.36 | 0.28 | -0.24 | .808 |
| Drink no more than usual × MB score | 0.88 | 0.12 | -0.41 | 0.15 | -0.90 | .368 |
| Drink less than usual × MB score | 0.71 | 0.09 | -0.58 | -0.10 | -2.78 | **.005** |

*Notes*. The MB score was derived from logistic mixed-effects regression. ‘Drink less than usual’ and ‘drink no more than usual’ refer to dummy-coded drinking intention, comparing the probability of drinking on days with this intention to the probability of drinking on days with ‘no specific intention’ (reference level), respectively. LL, lower limit; MB, model-based; *SE*, standard error of the mean; UP, upper limit; 95% CI, two-sided 95% Wald confidence interval

**Table S24.** Logistic mixed-effects regression model predicting the probability of drinking (vs. abstinence) on a given day in *n*=67 participants with alcohol use disorder by weekly drinking intentions, model-based behavior (MB score) in the two-step task, and model-based neural signatures

| **Fixed effect** | ***Odds Ratios*** | ***SE*** | **95% CI** | | ***z*-value** | ***p*** |
| --- | --- | --- | --- | --- | --- | --- |
|  |  |  | **LL** | **UL** |  |  |
| Intercept | 1.64 | 0.49 | -0.09 | 1.08 | 1.66 | .097 |
| Drink no more than usual | 1.25 | 0.33 | -0.29 | 0.73 | 0.85 | .393 |
| Drink less than usual | 0.82 | 0.19 | -0.64 | 0.26 | -0.85 | .397 |
| MB score | 0.95 | 0.16 | -0.37 | 0.27 | -0.29 | .770 |
| MB signatures hippocampus | 1.003 | 0.62 | -1.20 | 1.21 | 0.01 | .996 |
| MB signatures VS | 0.74 | 0.27 | -1.02 | 0.43 | -0.80 | .420 |
| MB signatures vmPFC | 0.75 | 0.30 | -1.09 | 0.51 | -0.72 | .473 |
| Drink no more than usual × MB score | 0.90 | 0.13 | -0.38 | 0.17 | -0.75 | .452 |
| Drink less than usual × MB score | 0.72 | 0.09 | -0.57 | -0.09 | -2.75 | **.006** |
| Drink no more than usual × MB signatures hippocampus | 0.61 | 0.30 | -1.46 | 0.47 | -1.00 | .315 |
| Drink less than usual × MB signatures hippocampus | 0.45 | 0.19 | -1.64 | 0.05 | -1.85 | .065 |
| Drink no more than usual × MB signatures VS | 1.93 | 0.60 | 0.05 | 1.26 | 2.11 | **.035** |
| Drink less than usual × MB signatures VS | 1.73 | 0.44 | 0.05 | 1.05 | 2.15 | **.031** |
| Drink no more than usual × MB signatures vmPFC | 0.87 | 0.29 | -0.79 | 0.51 | -0.43 | .669 |
| Drink less than usual × MB signatures vmPFC | 1.16 | 0.34 | -0.43 | 0.73 | 0.50 | .617 |

*Notes*. The MB score was derived from logistic mixed-effects regression. ‘Drink less than usual’ and ‘drink no more than usual’ refer to dummy-coded drinking intention, comparing the probability of drinking on days with this intention to the probability of drinking on days with ‘no specific intention’ (reference level), respectively. LL, lower limit; MB signatures, blood oxygenation level dependent signal associated with unique model-based reward prediction error components according to Daw et al. ^4^; *SE*, standard error of the mean; UP, upper limit; vmPFC, ventromedial prefrontal cortex; VS, ventral striatum; 95% CI, two-sided 95% Wald confidence interval

Supplementary references

1 Heinz A, Kiefer F, Smolka MN, Endrass T, Beste C, Beck A *et al.* Addiction research consortium: Losing and regaining control over drug intake (ReCoDe)—From trajectories to mechanisms and interventions. *Addiction Biology* 2020; **25**: e12866.

2 Beesdo-Baum K, Zaudig M, Wittchen H-U (eds). *Strukturiertes Klinisches Interview für DSM-5®-Störungen – Klinische Version*. 1st edn Hogrefe, 2019.

3 American Psychiatric Association. *Diagnostic and statistical manual of mental disorders*. 5th edn American Psychiatric Association: Washington, DC, 2013.

4 Daw ND, Gershman SJ, Seymour B, Dayan P, Dolan RJ. Model-based influences on humans’ choices and striatal prediction errors. *Neuron* 2011; **69**: 1204–1215.

5 Kool W, Cushman FA, Gershman SJ. When does model-based control pay off? *PLOS Computational Biology* 2016; **12**: e1005090.

6 Pelli DG. The VideoToolbox software for visual psychophysics: Transforming numbers into movies. *Spatial Vision* 1997; **10**: 437–442.

7 Brainard DH. The Psychophysics Toolbox. *Spatial Vision* 1997; **10**: 433–436.

8 Kleiner M, Brainard DH, Pelli DG. What’s new in Psychtoolbox-3? In: *Perception 36 ECVP Abstract Supplement*. 2007https://pure.mpg.de/pubman/faces/ViewItemFullPage.jsp?itemId=item_1790332.

9 Jaeger J. Digit Symbol Substitution Test: The Case for Sensitivity Over Specificity in Neuropsychological Testing. *J Clin Psychopharmacol* 2018; **38**: 513–519.

10 John, Raven J. Raven Progressive Matrices. In: McCallum RS (ed). *Handbook of Nonverbal Assessment*. Springer US: Boston, MA, 2003, pp 223–237.

11 Ramsay MC, Reynolds CR. Separate Digits tests: A brief history, a literature review, and a reexamination of the factor structure of the test of memory and learning (TOMAL). *Neuropsychol Rev* 1995; **5**: 151–171.

12 Saunders JB, Aasland OG, Babor TF, De la Fuente JR, Grant M. Development of the Alcohol Use Disorders Identification Test (AUDIT): WHO collaborative project on early detection of persons with harmful alcohol consumption-II. *Addiction* 1993; **88**: 791–804.

13 Sutton RS, Barto AG. *Reinforcement learning: An introduction*. Second edition. The MIT Press: Cambridge, Massachusetts, 2018.

14 Lau B, Glimcher PW. Dynamic response-by-response models of matching behavior in rhesus monkeys. *J Exp Anal Behav* 2005; **84**: 555–579.

15 Stan Development Team. Reference manual. 2023.https://mc-stan.org/.

16 Stan Development Team. RStan: The R interface to Stan. 2023.https://mc-stan.org/ (accessed 16 Feb2023).

17 R Core Team. R: A Language and environment for statistical computing. 2024.https://www.R-project.org/.

18 Ahn W-Y, Krawitz A, Kim W, Busmeyer JR, Brown JW. A model-based fMRI analysis with hierarchical bayesian parameter estimation. *J Neurosci Psychol Econ* 2011; **4**: 95–110.

19 Ahn W-Y, Haines N, Zhang L. Revealing neurocomputational mechanisms of reinforcement learning and decision-making with the hBayesDM package. *Comput Psychiatr* 2017; **1**: 24–57.

20 Vehtari A, Gelman A, Simpson D, Carpenter B, Bürkner P-C. Rank-normalization, folding, and localization: An improved Rˆ for assessing convergence of MCMC (with discussion). *Bayesian Analysis* 2021; **16**: 667–718.

21 Vehtari A, Gelman A, Gabry J. Practical Bayesian model evaluation using leave-one-out cross-validation and WAIC. *Stat Comput* 2017; **27**: 1413–1432.

22 Gillan CM, Kosinski M, Whelan R, Phelps EA, Daw ND. Characterizing a psychiatric symptom dimension related to deficits in goal-directed control. *eLife* 2016; **5**: e11305.

23 Voon V, Derbyshire K, Rück C, Irvine MA, Worbe Y, Enander J *et al.* Disorders of compulsivity: A common bias towards learning habits. *Mol Psychiatry* 2015; **20**: 345–352.

24 Smittenaar P, FitzGerald THB, Romei V, Wright ND, Dolan RJ. Disruption of dorsolateral prefrontal cortex decreases model-based in favor of model-free control in humans. *Neuron* 2013; **80**: 914–919.

25 Akam T, Costa R, Dayan P. Simple plans or sophisticated habits? State, transition and learning interactions in the two-step task. *PLOS Computational Biology* 2015; **11**: e1004648.

26 Friston KJ, Williams S, Howard R, Frackowiak RSJ, Turner R. Movement-related effects in fMRI time-series. *Magnetic Resonance in Medicine* 1996; **35**: 346–355.
